# Hunchback is counter-repressed to regulate *even-skipped* stripe 2 expression in Drosophila embryos

**DOI:** 10.1371/journal.pgen.1007644

**Published:** 2018-09-07

**Authors:** Ben J. Vincent, Max V. Staller, Francheska Lopez-Rivera, Meghan D. J. Bragdon, Edward C. G. Pym, Kelly M. Biette, Zeba Wunderlich, Timothy T. Harden, Javier Estrada, Angela H. DePace

**Affiliations:** Department of Systems Biology, Harvard Medical School, Boston, Massachusetts, United States of America; UNITED STATES

## Abstract

Hunchback is a bifunctional transcription factor that can activate and repress gene expression in *Drosophila* development. We investigated the regulatory DNA sequence features that control Hunchback function by perturbing enhancers for one of its target genes, *even-skipped (eve)*. While Hunchback directly represses the *eve* stripe 3+7 enhancer, we found that in the *eve* stripe 2+7 enhancer, Hunchback repression is prevented by nearby sequences—this phenomenon is called counter-repression. We also found evidence that Caudal binding sites are responsible for counter-repression, and that this interaction may be a conserved feature of *eve* stripe 2 enhancers. Our results alter the textbook view of *eve* stripe 2 regulation wherein Hb is described as a direct activator. Instead, to generate stripe 2, Hunchback repression must be counteracted. We discuss how counter-repression may influence *eve* stripe 2 regulation and evolution.

## Author summary

During development, animals express genes in distinct patterns to specify different cell types that are important for function. Enhancers are DNA sequences that generate these patterns by binding proteins that activate gene expression, repress gene expression, or both. We have learned how enhancers generate patterns by carefully studying particular DNA sequences, including the *even-skipped* stripe 2 enhancer, which generates expression in a single stripe in fruit fly embryos; this enhancer has been an important case study for the field. Although the protein Hunchback has been thought to activate this enhancer, we find that it actually functions as a repressor, and this repression is counteracted by nearby sequences in the enhancer. Our results change how we think the *even-skipped* stripe 2 enhancer works and evolves over time.

## Introduction

Bifunctional transcription factors (TFs) that can activate or repress their target genes are critical in animal development [1,2] and are associated with human disease [3,4]. The function of these TFs can depend on the context of the enhancer sequences they bind [5–9]. To infer accurate regulatory networks from genome sequence, we must define the sequence features that control the activity of bifunctional TFs [10].

Hunchback (Hb) is a bifunctional TF that patterns the *Drosophila melanogaster* embryo [11]; the Hunchback homolog Ikaros is critical in human hematopoesis [12]. *hb* is a gap gene with many targets throughout Drosophila development including other gap genes [13], pair-rule genes [14–17], homeotic genes [18–20] and neuronal genes [21]. Its bifunctional role in regulating the pair-rule gene *even-skipped* (*eve*) has been particularly well-studied. Classic experiments using minimized enhancer constructs found evidence that Hb directly activates the minimal *eve* stripe 2 enhancer (*eve2min*) [14,22,23]. Other studies examined endogenous *eve* expression by misexpressing *hb* along the ventral surface of embryos (*sna*::*hb* embryos) and found that Hb represses *eve* stripes 3 and 7 [24]. Qualitative measurements of mutated versions of the *eve* stripe 3+7 enhancer (*eve3+7*) driving reporter gene expression suggested that Hb repression of *eve3+7* is direct [15,17,25,26].

Even after decades of study, the DNA sequence features that control Hb bifunctionality are not known. One hypothesis is that DNA-bound Hb dimers act as repressors while Hb monomers act as activators (dimerization hypothesis). The dimerization hypothesis is supported by computational work that predicts expression of *eve3+7* and the gap gene *Krüppel* [27,28], as well as *in vitro* experiments identifying zinc finger domains in Hb and Ikaros that allow for dimerization [29]. In another hypothesis, binding of a different TF converts Hb from a repressor into an activator (co-activation hypothesis). *In vivo* measurements of synthetic binding site arrays for Bicoid (Bcd) and Hb support the co-activation hypothesis [30], and Hb co-activation by Bcd has been incorporated into computational models of *eve* enhancer function [31–33]. Recent models have also incorporated Hb co-activation by the TF Caudal (Cad) [34]. Cad activates gap and pair-rule gene expression in the posterior of both long and short germ-band insects [35–38], and Cad homologs are critical in vertebrate development and human disease [39,40]. Though both hypotheses have been included in computational models of enhancer function [28,34], neither has been experimentally tested.

**Fig 1:**
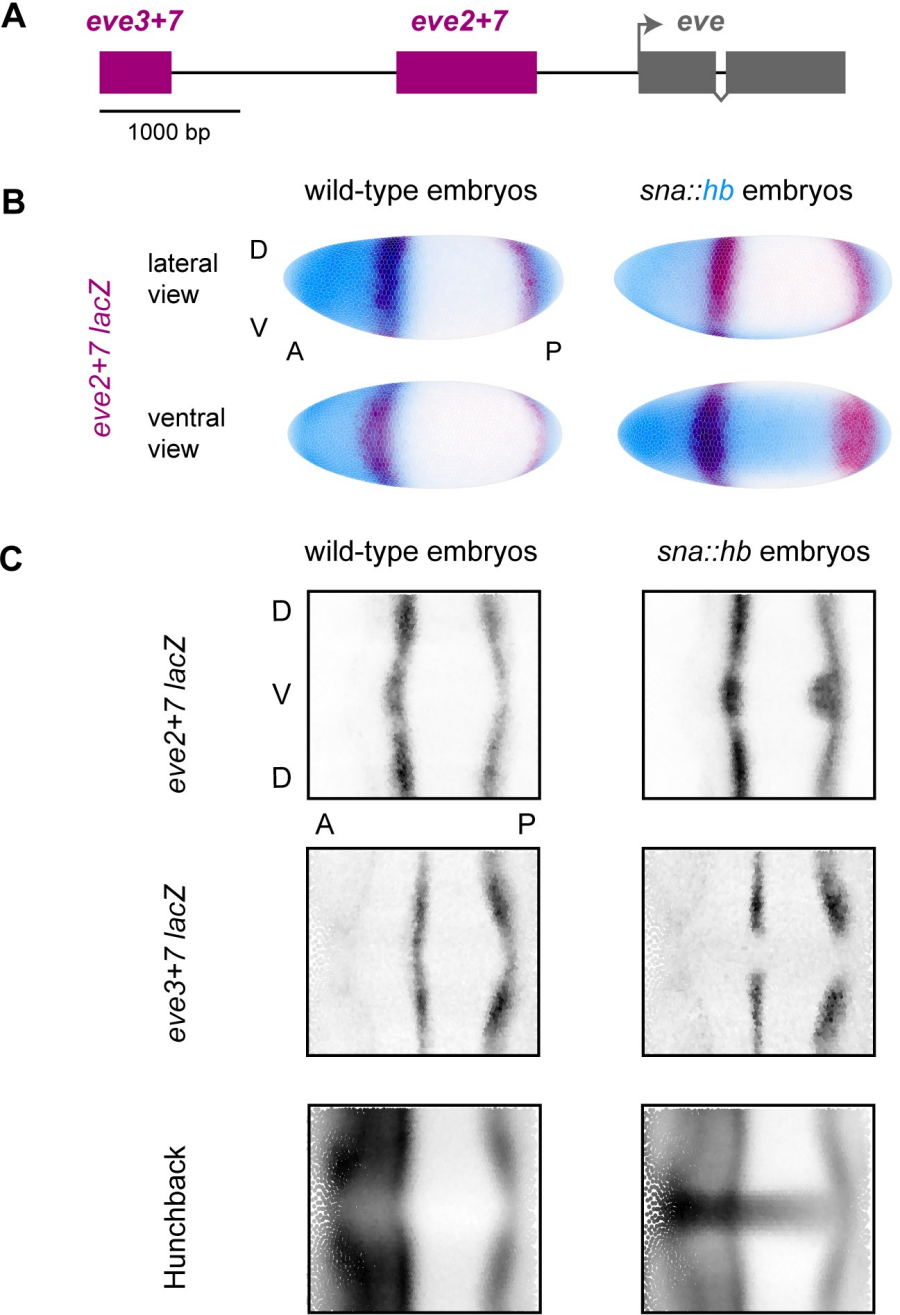
Quantitative gene expression data in wild-type and *sna*::*hb* embryos. (A) Diagram of the upstream half of the *even-skipped* locus. Enhancers are indicated in maroon, and coding sequence is indicated in grey. (B) We created transgenic lines containing *lacZ* reporter constructs for *eve* enhancers and measured gene expression in wild-type embryos and embryos misexpressing ventral *hb* (*sna*::*hb* embryos). Here, we show visual renderings [74] of gene expression atlas data that average measurements from multiple embryos in timepoint 4 (25–50% membrane invagination) [43]. Dorsal (D) and ventral (V) surfaces are indicated for the lateral view, as are anterior (A) and posterior (P) positions. Left: *eve2+7 lacZ* expression (maroon) in wild-type embryos; Right: *eve2+7 lacZ* in *sna*::*hb* embryos. Hb protein is shown in blue. Individual nuclei are outlined, and darker coloring indicates higher relative expression level. (C) To help visualize all relevant nuclei, we show 2-dimensional projections of expression data throughout this manuscript. Positions of individual nuclei along the dorsal-ventral (DV) axis are plotted as a function of position along the anterior-posterior (AP) axis. Darker color indicates higher expression for each nucleus. Relative expression values are normalized to the maximum value and range from 0 to 1.

Here, we experimentally test the co-activation hypothesis by perturbing two enhancers that are active in the same cells but respond differently to Hb. *eve* stripe 7 is generated by two shadow enhancers; Hb represses *eve3+7* [24,26] and activates *eve2+7*, an extended version of *eve2min* that also generates stripe 7 (Fig 1A and 1B) [41]. Because both enhancers are active in the same nuclei, Hb function must be partially controlled by enhancer sequence. In this system, we can test the co-activation hypothesis by measuring the effects of perturbing both enhancers quantitatively and at cellular resolution (Fig 1) [42–44].

We find that Hb directly represses *eve3+7* and indirectly activates *eve2+7*. Indirect Hb activation occurs because nearby sequences corresponding to predicted Cad binding sites prevent Hb repression in *eve2+7*. We also find that the number of predicted Cad and Hb sites appears correlated in orthologous *eve* stripe 2 enhancers. Our results suggest that the textbook description of *eve2min*, where Hb is described as a direct activator that synergizes with Bcd [45], is incomplete. Instead, Hb represses *eve* stripe *2*, but this activity is inhibited by additional factors.

## Results

### Hb directly represses *eve3+7*

Previous work indicated that Hb directly represses *eve3+7*, but did not decipher how Hb regulates expression of each stripe. For example, mutating Hb binding sites in *eve3+7* showed that Hb defines the anterior border of stripe 3, but the effect of Hb on stripe 7 was unclear [26]. Furthermore, the stripe 7 pattern driven by *eve3+7* retreats in response to ectopic Hb, but this effect could be indirect [41]. Here, we combined perturbations in both enhancer sequence and Hb protein localization and measured the effects on expression of each stripe quantitatively.

**Fig 2:**
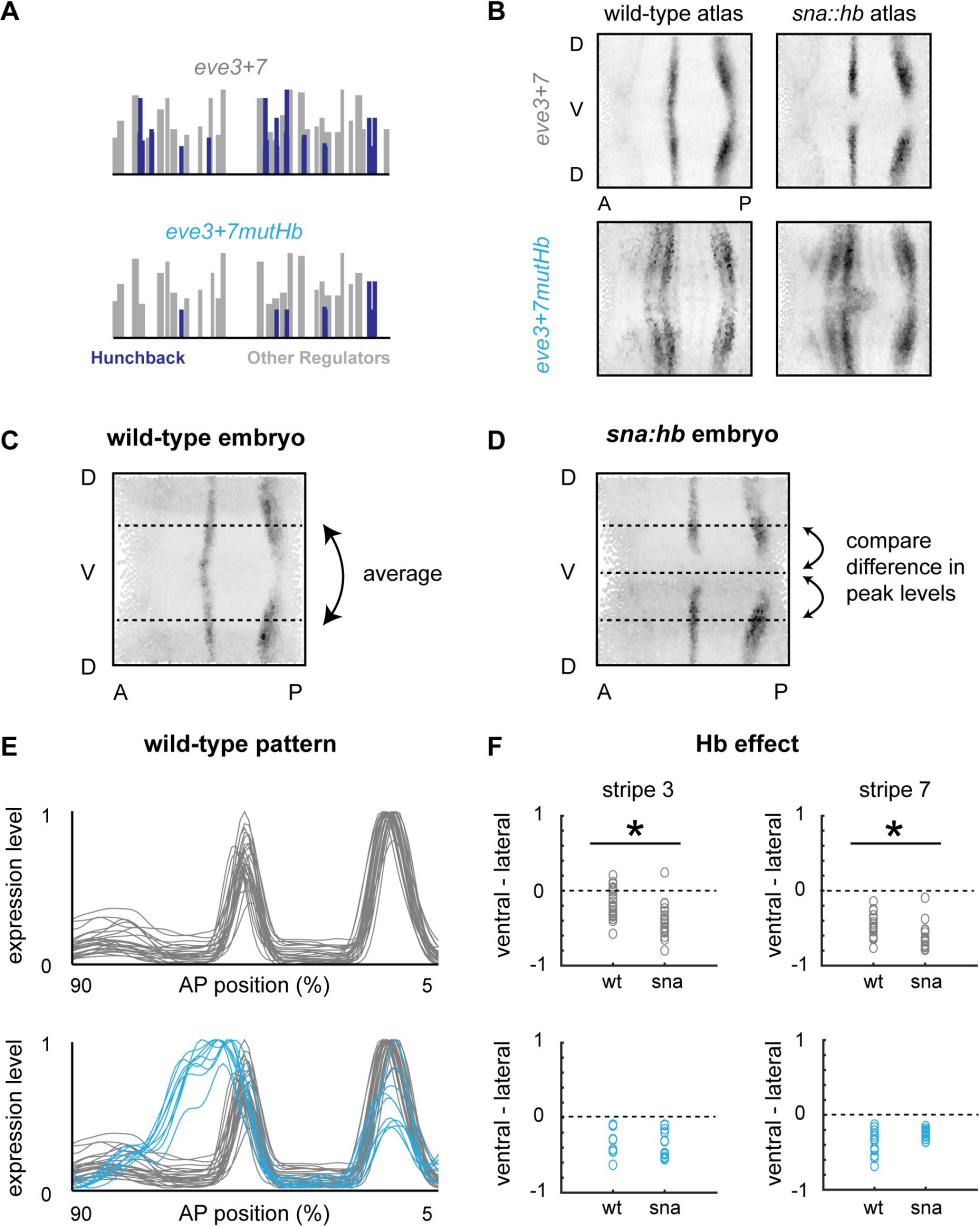
Hunchback directly represses *eve3+7*. (A) Predicted Hb binding sites in wild-type and mutant versions of *eve3+7* are depicted as vertical bars along the sequence where height is proportional to PATSER score [68]. Hb sites are indicated in blue; other regulators are in grey. (B) 2D projections of atlas data for reporter constructs expressed in wild-type or sna::hb embryos. Data is taken from timepoint 4. (C) 2D projection of a representative wild-type embryo expressing *eve3+7 lacZ*. We plot gene expression as a function of anterior-posterior (AP) position by averaging measurements from lateral strips in individual embryos and normalizing them to their maximum value (see E). (D) 2D projection of a representative *sna*::*hb* embryo expressing *eve3+7 lacZ*. To quantify the effect of ventral Hb in individual embryos, we take average measurements from lateral strips, extract the local maxima corresponding to stripes 3 and 7, and subtract those values from the corresponding peaks of the ventral strip. We perform the same analysis in wild-type embryos to account for modulation along the DV axis. A decrease in the ventral/lateral difference between wild-type and *sna*::*hb* embryos indicates Hb repression, while an increase indicates Hb activation (see F). (E) Lateral line traces from individual wild-type embryos containing *eve3+7* reporter constructs (*eve3+7*: grey, n = 26; *eve3+7mutHb*: blue, n = 9). Embryos are from all six timepoints in stage 5. (F) Differences between ventral and lateral stripe peaks are plotted for individual wild-type and *sna*::*hb* embryos in all 6 timepoints in stage 5. Top: wild-type *eve3+7* (wt: n = 26; *sna*::*hb*: n = 19); Bottom: *eve3+7mutHb* (wt: n = 9; *sna*::*hb*: n = 13). Asterisks indicate significant differences between wild-type and *sna*::*hb* embryos (p-value < 0.001, Mann-Whitney U test). Differences between wild-type and *sna*::*hb* embryos containing *eve3+7mutHb* were not significant (p-value > 0.4 for both stripes, Mann-Whitney U test).

We removed predicted Hb binding sites from *eve3+7* (*eve3+7mutHb*) using the most current position weight matrices (PWMs) available; PWMs allow us to predict TF binding at a variety of different stringencies (see Materials and Methods). Some predicted low affinity Hb sites remain because they overlap with sites for other factors (Fig 2A, S1 Appendix). We incorporated *eve3+7mutHb* into a reporter construct, integrated it into the *Drosophila melanogaster* genome and measured expression using quantitative *in situ* hybridization in wild-type and *sna*::*hb* embryos, where Hb protein is produced along the ventral side of the embryo. We compared expression driven by this construct to expression driven by a wild-type version (Fig 2). We also measured expression driven by a mutant construct from [26] (S1 Fig). We display our quantitative gene expression data in multiple ways: a line trace of expression level versus anterior-posterior (AP) position along the lateral surfaces of the embryo (Fig 2C and 2E); a 2-dimensional rendering of the expression level in every cell in a single embryo (Fig 2C and 2D) or gene expression atlas (Fig 2B); or a plot of the differences in peak expression level between a lateral and a ventral line trace in individual *sna*::*hb* embryos to quantify the effect of *hb* misexpression (Fig 2D and 2F). Means and standard errors for our quantitative measurements can be found in S1 and S2 Tables. All enhancer sequences in this study were incorporated into the same reporter backbone and integrated into the same genomic location (see Materials and Methods). Annotated *eve3+7* and *eve2+7* enhancer sequences from this study are included in S1 Appendix.

Consistent with previous results [26], *eve3+7mutHb* drove an expression pattern where stripe 3 expanded anteriorly, though we detected no effect on the boundaries of stripe 7 (Fig 2B and 2E). In addition, the stripe 7 expression pattern driven by *eve3+7mutHb* did not retreat from ventral Hb in *sna*::*hb* embryos (Fig 2B and 2F). By combining perturbations to enhancer sequence and Hb protein localization, these results confirm that Hb directly represses *eve3+7*.

### Hb indirectly activates stripe 7 in *eve2+7*

**Fig 3:**
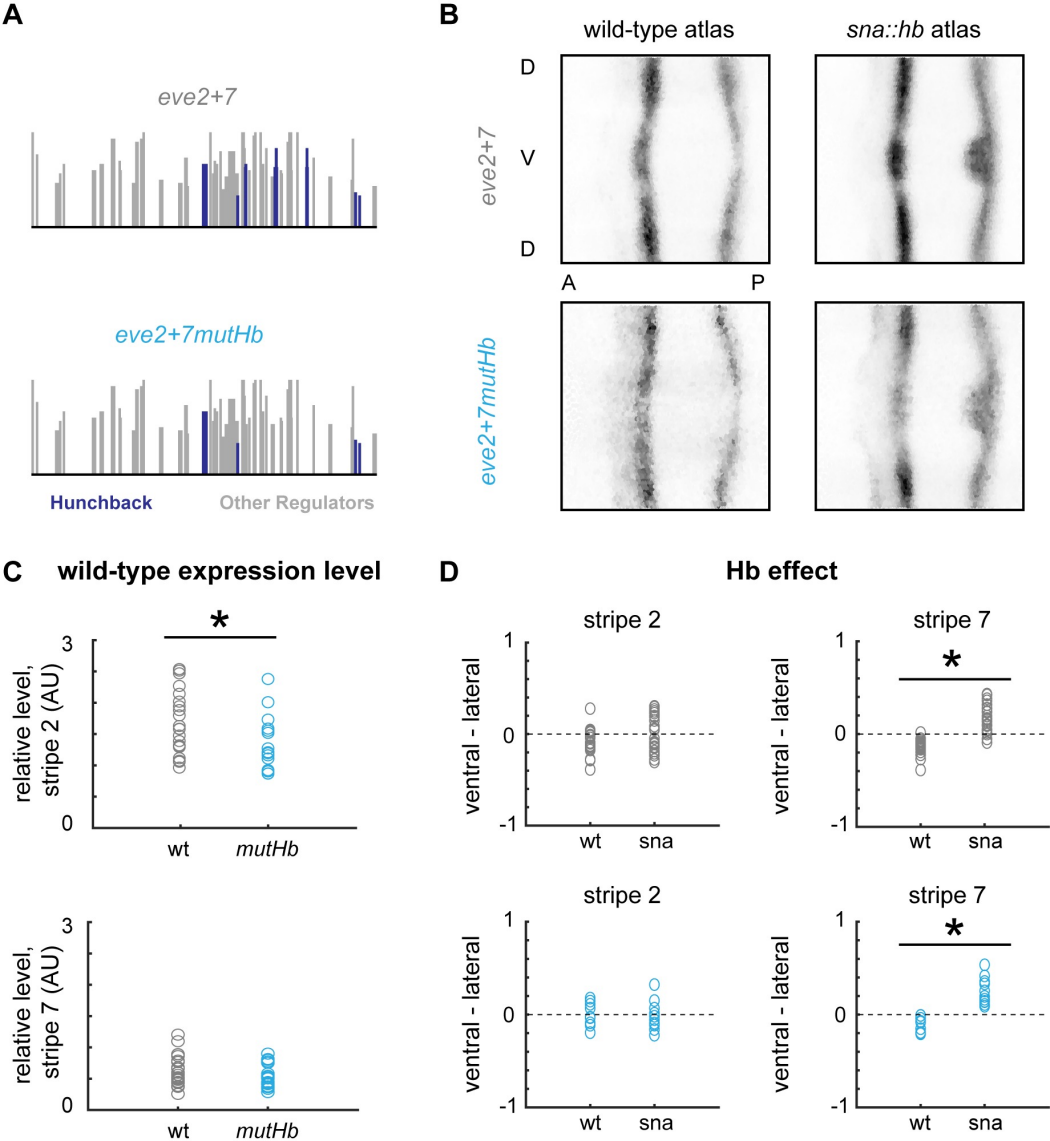
Hunchback indirectly activates *eve2+7*. (A) Predicted Hb binding sites in *eve2+7* and *eve2+7mutHb*. Sites are displayed as in Fig 2. (B) 2D projections of atlas data for reporter constructs expressed in wild-type or *sna*::*hb* embryos. Data is taken from timepoint 4. (C) Peak stripe expression levels for individual embryos from timepoints 2–4 (4–50% membrane invagination) containing *eve2+7* (grey, n = 23) or *eve2+7mutHb* (blue, n = 20) were measured by normalizing *lacZ* expression levels using a *huckebein* co-stain [44] and extracting local maxima from lateral line traces. Asterisks indicate significant differences (p-value < 0.05, Mann-Whitney U test). (D) Differences between ventral and lateral stripe peaks are plotted for individual wild-type and *sna*::*hb* embryos in all 6 timepoints in stage 5. Top: wild-type *eve2+7* (wt: n = 22; *sna*::*hb*: n = 26); Bottom: *eve2+7mutHb* (wt: n = 9; *sna*::*hb*: n = 11). Asterisks indicate significant differences between wild-type and *sna*::*hb* embryos (p-value < 0.001, Mann-Whitney U test).

Previous experimental and computational work suggested that Hb directly activates *eve* stripe *2* [23,31]. We previously found that the stripe 7 pattern driven by *eve2+7* bulges in *sna*::*hb* embryos, and thus hypothesized that Hb directly activates *eve2+7* [41]. Here, we tested whether Hb directly activates stripe 7 in *eve2+7* by mutating predicted Hb binding sites in *eve2+7* (*eve2+7mutHb*, Fig 3A) and measuring gene expression in wild-type and *sna*::*hb* embryos.

We were surprised to find that mutating Hb binding sites had little effect on the spatial pattern driven by *eve2+7* in either genetic background. Compared to *eve2+7*, *eve2+7mutHb* drives low level expression anterior to stripe 2 (Fig 3B; line traces for individual embryos are shown in S2 Fig), but other features of the expression pattern were unaffected, including the stripe 7 bulge in *sna*::*hb* embryos. We therefore tested for quantitative effects of removing Hb binding sites by measuring expression levels using a co-stain method [44]. We found that *eve2+7mutHb* drove slightly lower expression levels in stripe 2 (p-value = 0.022, Mann-Whitney U test), but stripe 7 levels were indistinguishable between *eve2+7* and *eve2+7mutHb* (p-value = 0.304, Mann-Whitney U test) (Fig 3C).

Given the subtle effects that Hb binding site mutations had in *eve2+7*, we examined the behavior of other *eve* stripe 2 enhancer fragments in the literature (see S2 Appendix for annotated enhancer sequences). The *eve* stripe 2 fragment used in comparative work is smaller than *eve2+7* by 100 base pairs on either side, but contains all three binding sites that we mutated in *eve2+7mutHb [46]*. When we mutated these binding sites in this fragment, we observed a significant decrease in stripe 2 expression level (S3 Fig). The minimal *eve* stripe 2 enhancer (*eve2min*) is smaller still and contains only one of the sites mutated in *eve2+7mutHb*. Previous work using qualitative methods has suggested that mutating this binding site decreases expression driven by *eve2min* [23,47], which has been central to cartoon models of *eve* stripe 2 in textbooks [45] and computational models of enhancer function in the field [31,34]. However, using our quantitative methods, we observed no significant decrease in expression level as a result of mutating this Hb binding site in *eve2min* (S3 Fig). These data indicate that Hb binding site mutations in *eve* stripe 2 enhancers have small, context-dependent effects on gene expression. Taken together, our results further suggest that while Hb may be a weak activator of stripe 2, it does not influence stripe 7 expression at endogenous Hb levels in either *eve3+7* or *eve2+7*.

### Mutations in predicted Caudal binding sites prevent repression of *eve2+7* by Hb

**Fig 4:**
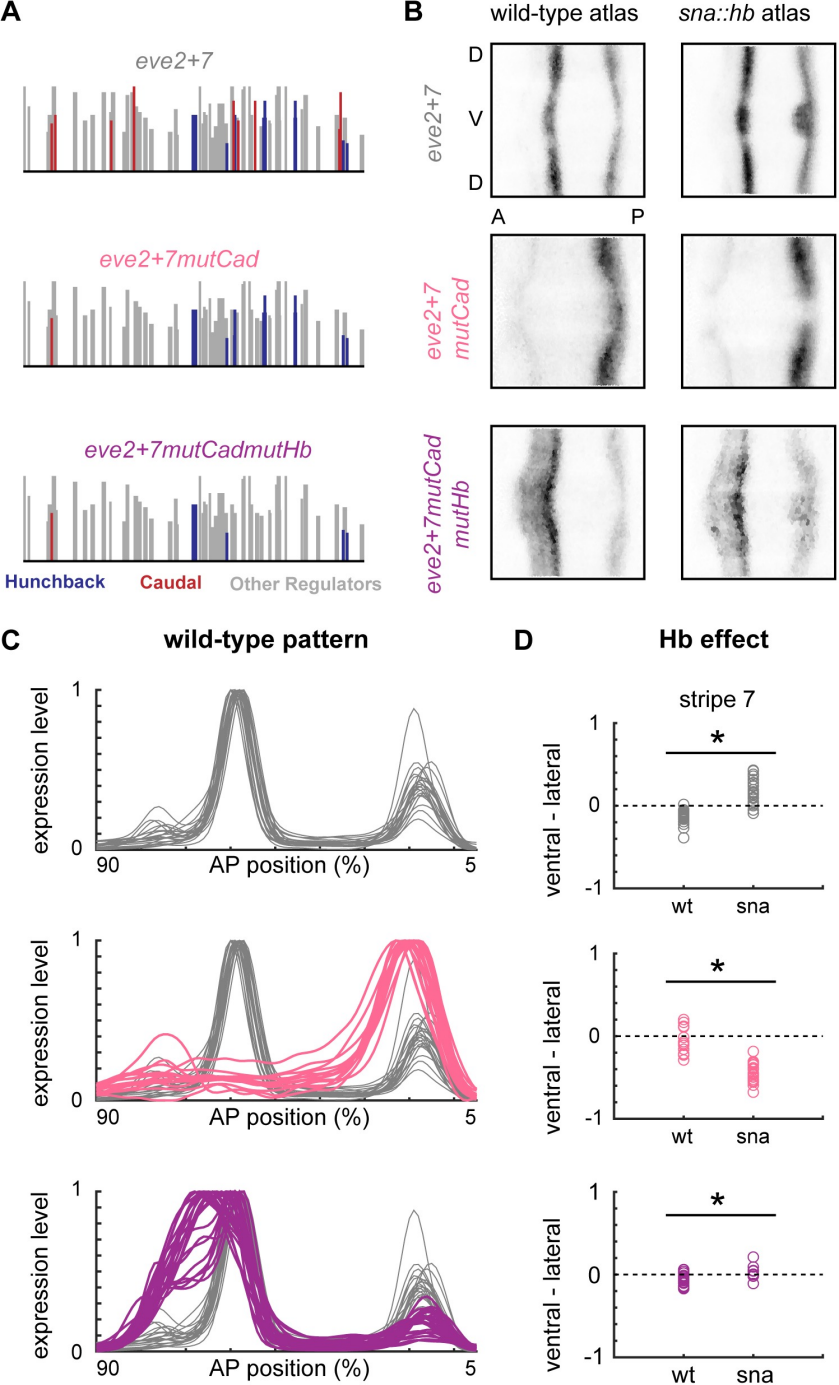
Hunchback is counter-repressed in *eve2+7*. (A) Predicted Cad (red) and Hb (blue) binding sites in wild-type and mutant versions of *eve2+7* are shown as in Fig 2. (B) 2D projections of atlas data for reporter constructs expressed in wild-type or *sna*::*hb* embryos. Data is taken from timepoint 4. (C) Lateral line traces from individual wild-type embryos containing *eve2+7* reporter constructs. Embryos are from all six timepoints in stage 5. (D) Differences between ventral and lateral stripe peaks are plotted for individual wild-type and *sna*::*hb* embryos in all 6 timepoints in stage 5. Top: wild-type *eve2+7* (wt: n = 22; sna: n = 26); Middle: *eve2+7mutCad* (wt: n = 15; sna: n = 24); Bottom: *eve2+7mutCadmutHb* (wt: n = 22; sna: n = 9). Asterisks indicate significant differences between wild-type and *sna*::*hb* embryos (p-value < 0.001, Mann-Whitney U test).

Our data indicate that Hb directly represses *eve3+7* but has little effect in *eve2+7*. One possible explanation for this result is that Hb functions as a weak activator in *eve2+7*. If this is the case, then the hypothesis that Hb acts as an activator by interacting with Cad (the co-activation hypothesis) makes a strong prediction: mutating Cad sites in *eve2+7* should convert Hb from a weak activator to a repressor of that sequence. We tested this hypothesis by mutating predicted Cad binding sites in *eve2+7* (*eve2+7mutCad*, Fig 4A), and measuring gene expression in wild-type and *sna*::*hb* embryos. These mutations abolished stripe 2 expression completely and caused an anterior expansion of stripe 7 (Fig 4B and 4C). We hypothesize that this anterior expansion of stripe 7 was caused by unintended mutations in binding sites for the repressor Giant (Gt), whose predicted binding sites overlap with Cad (S4 Fig). In *sna*::*hb* embryos, removing Cad binding sites caused the stripe 7 pattern to retreat (Fig 4B and 4D). This result suggests that without Cad, Hb behaves as a repressor in this sequence. To confirm that this effect was due to direct repression by Hb, we mutated both Hb and Cad binding sites in *eve2+7* (*eve2+7mutCadmutHb*, Fig 4A). We found that removing predicted Cad and Hb binding sites restored the stripe 2 expression that was lost following the removal of predicted Cad sites alone (Fig 4B and 4C). We also observed anterior expansions of stripe 2 and stripe 7; we attribute this effect to the unintended loss of a binding site for Gt, as mentioned above (see Discussion). Most importantly, these additional Hb mutations abolished repression by ventral Hb (Fig 4B and 4D). These results therefore support a variation of the co-activation hypothesis where Cad prevents Hb from directly repressing *eve2+7*.

If Cad binding to *eve2+7* prevents Hb repression, removing Cad from the embryo should result in the loss of *eve* stripe 2 expression. However, previous work examining endogenous *eve* expression in Cad mutant embryos did not describe any effect on stripe 2 [38]. We therefore attempted to knock-down *cad* expression in embryos by expressing short hairpin RNAs targeting *cad* (*cad* RNAi embryos). However, we observed no qualitative changes in endogenous *eve* expression in *cad* RNAi embryos **(**S5 Fig**)**, suggesting a low knock-down efficiency of our RNAi method. As *cad* is both maternally and zygotically expressed [35,36,48,49], these results are consistent with previous observations that this method is effective at depleting maternally deposited transcripts, but not transcripts that are zygotically expressed in the early embryo [50]. We therefore cannot rule out the possibility that some sequence feature other than Cad binding sites controls Hb activity (see Discussion).

### Predicted Hunchback and Caudal binding sites co-evolve in orthologous *eve* stripe 2 enhancers

**Fig 5:**
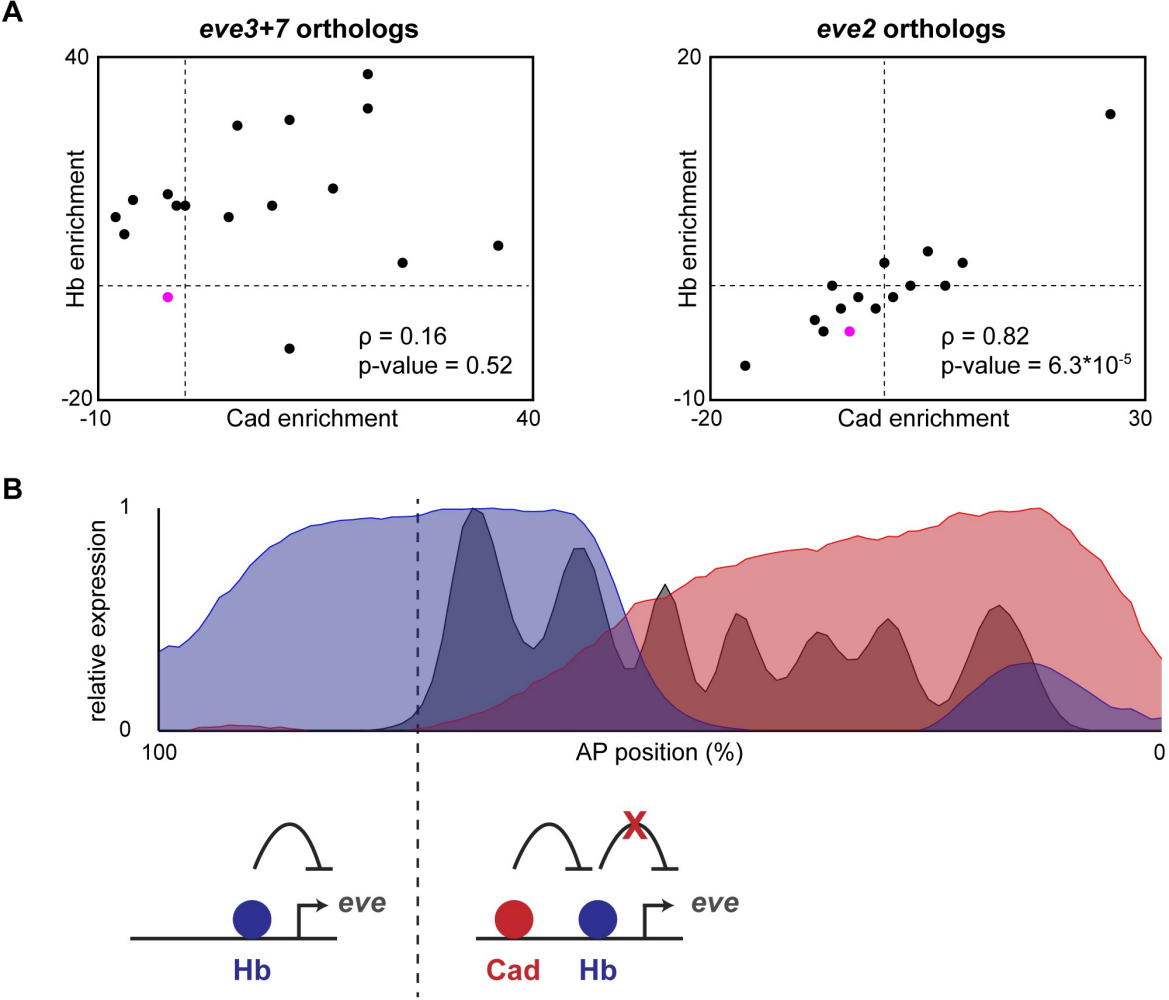
Caudal and Hunchback binding sites co-evolve in orthologous eve stripe 2 enhancers. (A) Enrichment scores for predicted Hb and Cad binding sites in orthologous *eve* stripe *2* and *eve3+7* sequences from different Drosophilid and Sepsid species [51]. Scores were calculated by comparing the number of predicted sites to an expected value calculated from the genomic background [52]. Scores for *Drosophila melanogaster* enhancers are indicated in magenta. Spearman correlation and p-value are displayed for each set of enhancers. (B) Summary of current findings. Top: levels of Hb protein (blue), Cad protein (red) and Eve protein (grey) are plotted as a function of anterior-posterior position. Data were taken from the FlyEx database [75]. Bottom: cartoon indicating Hb function in *eve2+7* at different locations in the embryo. In the anterior, Cad levels are low, so Hb represses *eve2+7*. In the trunk, Cad binding to *eve2+7* prevents Hb repression.

Our results suggest that predicted Cad sites in *eve2+7* prevent Hb from repressing *eve* stripe 2. We therefore hypothesized that the balance between Hb and Cad binding sites is under selective constraint, which would result in Hb and Cad binding sites co-evolving in *eve* stripe 2 orthologs. To test this hypothesis, we predicted Cad and Hb binding sites in orthologous *eve* stripe *2* enhancers previously identified in Drosophilid and Sepsid genomes [51], and calculated how much they were enriched compared to other regions of open chromatin during stage 5 of embryogenesis in *Drosophila melanogaster* [52]. We found that Cad and Hb enrichment scores were significantly correlated in orthologous *eve* stripe 2 enhancers (Fig 5A). In contrast, we did not observe any correlation between Hb and Cad binding sites in orthologous *eve3+7* enhancers when using the same PATSER score threshold used for predicted binding sites (Fig 5A). This result is notable since mutating Cad binding sites in *eve3+7* decreased expression of both stripes (S6 Fig), which suggests that Cad also directly activates *eve3+7*. However, we note that the correlation we observe in predicted sites depends on the PATSER score threshold used for identifying functional sites– at higher values for this threshold, we also observe a correlation in *eve3+7* orthologs (S7 Fig). Finally, we used the same method to test for a correlation in Hb and Bcd enrichment scores for *eve2* and *eve3+7* orthologs, but we observed no significant correlation in either case (S8 Fig). Taken together, these results suggest that counter-repression of Hb by Cad is a conserved feature of *eve* stripe 2 enhancers, although we cannot rule out the possibility that a similar interaction also occurs in *eve3+7* (see Discussion).

## Discussion

The *eve* enhancers are often used to teach the fundamental principles of patterning [45,53]. Hunchback (Hb) is a key regulator of *eve*; it is thought to activate the *eve* stripe 2 enhancer in concert with Bicoid (Bcd), while it acts as a repressor at other enhancers, such as *eve3+7* [24,26,54]. Here, we coupled quantitative imaging and systematic perturbations of regulatory DNA and TF expression to uncover the DNA sequence features that control Hb activity. We suggest that Hb activity is controlled by a second TF, Caudal (Cad), and discuss the implications for *eve* stripe 2 regulation and evolution.

### Revising our picture of eve stripe 2 regulation

In the textbook picture of *eve* stripe 2 [45], Bcd and Hb directly activate the enhancer in the anterior of the embryo, while Gt and Kr directly repress it to carve out a single stripe of expression [14,22,23]. However, attempts to reconstitute the minimal *eve* stripe 2 enhancer from its component binding sites have failed, which suggests that there is still more to learn about its regulatory logic [55]. For example, these interactions alone cannot explain how *eve* stripe 2 is repressed in the anterior tip of the embryo. Two other mechanisms have been proposed to account for this discrepancy: direct repression by Slp1 and downregulation of Bcd activity by Torso [56]. Our results suggest an additional mechanism: Hb may repress *eve2+7* in the anterior, possibly due to the absence of Cad (Fig 5B). In our data, *eve2+7mutHb* generates low level expression anterior to stripe 2 (Fig 3B, S2 Fig), which suggests that Hb may function with other factors in defining the anterior boundary of the stripe [22]. However, these results may also be due to mutations in binding sites for other factors, including Gt [46]. Indeed, we observe that mutating Hb and Cad binding sites in *eve2+7* causes a dramatic anterior expansion of both stripes, which we hypothesize is partially due to unintended mutations in Gt binding sites that overlap predicted Cad sites (see below).

Bcd and Hb have been proposed to synergistically activate *eve* stripe 2 [14,30], but our results do not support this hypothesis. Computational models that include Bcd/Hb synergy predict that Hb binding site mutations cause large decreases in expression [31]. However, our data suggest only modest effects of Hb binding site mutations in accordance with qualitative data in the literature [23,47]. In addition, our results do not support the previous model for Bcd/Hb synergy in *eve2+7*; without predicted Cad sites, Hb represses *eve2+7* even though Bcd binding sites are the same.

Hb has long been considered a critical activator of *eve* stripe 2, but our results suggest that its function is more complicated. First, mutating a footprinted Hb site in the context of the minimal *eve* stripe 2 enhancer did not affect expression level (S3 Fig), which directly contradicts previous qualitative data in the literature [23]. Mutating the same site and two others in the context of *eve2+7* causes a slight decrease in the expression of stripe 2 but not stripe 7 (Fig 3C). From these data, we see that three Hb binding sites in *eve2+7* may perform two functions with quantitatively small effects: weak activation of *eve* stripe 2 and repression of low-level expression anterior to *eve* stripe 2. Other Hb binding sites in *eve2+7* of lower predicted affinity may also contribute to these functions, but we did not mutate these sites to test this hypothesis. Because our findings rely on reporter constructs, it remains to be seen which quantitative features of *eve* stripe expression are necessary in the endogenous context for development to proceed properly. Thus, we cannot rigorously address whether the presence of Hb binding sites in *eve2+7* confers a selective advantage, but this question would be interesting to examine in future work.

### Discrepancies between Caudal perturbations in *cis* and *trans*

Developmental enhancers are complex, and dissecting their regulatory logic with either *cis* or *trans* perturbations carries caveats. Making sequence perturbations in *cis* can disrupt nearby or overlapping sites for known or unknown regulators. In addition, mutating or misexpressing regulators in *trans* can affect the location, level and function of other genes in the network [57]. These caveats are important to consider in this study, since endogenous *eve* stripe 2 is expressed in *cad* mutant embryos [38], which conflicts with our result that mutations in Cad binding sites in *eve2+7* abolish stripe 2 expression. While our binding site mutations may have disrupted other sequence features that are necessary for counter-repression, this discrepancy may be explained in other ways. First, *hb* expression may be affected in a *cad* mutant background. While *hb* expression is detectable in this background by qualitative *in situ* hybridization [38], *hb* expression levels may be lower, which may affect its capacity to repress stripe 2. Second, the piece of DNA that we tested in reporters may not contain all relevant DNA that contributes to *eve* stripe 2 expression. Indeed, in the endogenous *eve* locus, the intervening DNA between *eve2+7* and *eve3+7* contains binding sites for many *eve* regulators, but is notably devoid of predicted Hb binding sites [41]. Therefore, sequence adjacent to *eve2+7* may not be affected by Hb repression in the absence of *cad*. Finally, the placement of the *eve2+7* enhancer adjacent to the promoter in our reporter constructs may allow Hb to exert a stronger repressive effect than in the endogenous locus.

In this study, we combined *cis* perturbations to enhancer sequence and *trans* perturbation of upstream regulators to mitigate the drawbacks of each method. In our previous work, discrepancies between the *eve3+7* reporter pattern and the endogenous stripes revealed the influence of *eve2+7* as a stripe 7 shadow enhancer [41]; further dissection of stripe behavior in *cad* mutant embryos, using both reporter constructs and perturbations to the endogenous locus, may yield additional insights into *eve* regulatory logic.

### Counter-repression may influence regulatory evolution

Our results indicate that Hb binding sites in *eve2+7* can repress stripe 2. Previous work has shown that the absence of *eve* stripe 2 is lethal in *Drosophila melanogaster* [58]. It follows that selective mechanisms should operate to prevent repression of stripe 2 by Hb. One such mechanism could be selection against Hb sites in *eve2*. Indeed, we find that Hb binding sites are underrepresented in the *eve* stripe 2 enhancer sequence from *Drosophila melanogaster* (Fig 5A) [51]. Similarly, most *eve3+7* and *eve4+6* orthologs are depleted in binding sites for the repressor Kr, which overlaps stripes 3 and 4, while all *eve* stripe 2 enhancers are enriched in Kr binding sites, which are thought to define the posterior border of the stripe 2 pattern (S3 Table). These results suggest that selection may generally operate against binding sites for repressors that spatially overlap a developmental expression pattern.

However, our findings suggest that counter-repression may provide an additional mechanism that prevents repression of stripe 2 by Hb. We speculate that counter-repression may be useful, in that it allows for the intrusion of Hb sites over evolutionary time. Hb sites may arise because they play important functional roles in patterning the blastoderm embryo, or at other stages of development (e.g.[59]).

The correlation we observe between enrichment scores for predicted Cad and Hb sites in orthologous *eve* stripe 2 enhancers may reflect counter-repression in that enhancer specifically. However, this bioinformatic signature is not enough to rule out the possibility that Cad also counter-represses Hb in *eve3+7*. Sensitivity analyses indicate that the correlation of Hb and Cad binding sites in *eve* stripe *2* orthologs is highest when sites with lower predicted affinity (i.e. lower PATSER scores) are included, while in *eve3+7* orthologs we find that correlation values are highest when only sites of high predicted affinity are included (S7 Fig). These results may reflect differences in the constraints on the regulatory logic of *eve2+7* and *eve3+7*: for example, if Hb repression in *eve2+7* must be prevented or counteracted by Cad to allow for stripe 2 expression, whereas Hb repression in *eve3+7* is necessary for correct positioning of the stripes but may be modulated by Cad binding. Nonetheless, the co-evolutionary signature we observe in *eve* stripe *2* orthologs suggests that a functional interaction between Cad and Hb sites may be a conserved feature of *eve* stripe 2 regulation.

### Hb ‘bifunctionality’ is due to counter-repression

Our previous work indicated that Hb activates *eve2+7* and represses *eve3+7*. Because Hb was “known” to be an activator of *eve2min* and repressor of *eve3+7*, we hypothesized that these interactions were direct. Here, we find that Hb activation of *eve* stripe 7 is indirect. We now hypothesize that the *sna*::*hb* perturbation causes retreat of Gt and subsequent anterior expansion of the stripe 7 pattern [41]. Knirps forms the anterior boundary of stripe 7 through the *eve3+7* enhancer [26], while Gt has been proposed to form the anterior boundary of stripe 7 through *eve2+7* [31]. Indeed, we believe that the anterior stripe 7 expansion we observe after mutating predicted Cad sites in *eve2+7* is caused by unintended mutations in Gt binding sites. Predicted Gt sites overlap with predicted Cad sites in this sequence, and our mutations disrupt a Cad site and a predicted Gt site that has been confirmed by footprinting in *eve2min* (S4 Fig) [60]. It is challenging to mutate Cad sites without impacting Gt sites because measured binding preferences for Gt vary greatly between methods and do not exactly correspond to footprinted sites in the literature [22,61]. While these challenges make it difficult to determine whether Cad directly activates *eve2+7*, they do not affect our conclusions concerning Hb counter-repression. Indeed, the anterior expansion of stripe 7 makes the repressive influence of ventral Hb even more striking (Fig 4B and 4D).

Which TFs activate *eve* stripe 7 expression is less clear, though Cad and Zelda are obvious candidates shared by *eve2+7* and *eve3+7*. Overall, the conclusion of our previous work—that *eve* stripe 7 shadow enhancers use different regulatory logic—still holds [41]. Hb represses *eve3+7* but has little effect on *eve2+7* because of counter-repression. Furthermore, we hypothesize that the two enhancers use different repressors to set the anterior border, though this remains to be tested directly. We also previously showed that Hb activates and represses separate *Kr* shadow enhancers using the same *sna*::*hb* misexpression assay [52]; it is thus possible that Cad also counter-represses Hb in the proximal *Kr* enhancer.

Cad may counter-repress Hb through any number of molecular mechanisms. For example, Cad may interfere with Hb via direct protein-protein interactions. The simplest idea is that when Cad is present, Hb does not bind. This hypothesis is not supported by genome-wide chromatin immunoprecipitation (ChIP) data for both Hb and Cad, as both are bound to *eve2+7* [62,63]. We note two caveats of this data. First, it is not spatially resolved, so it remains possible that Hb binding is affected in a subset of cells. Second, Cad may disrupt Hb binding quantitatively, which may be enough to affect function but not enough to detect changes by ChIP. Another possibility is that direct protein-protein interactions between Cad and Hb interfere with Hb protein domains that execute its repressive function [21]. Finally, interactions could occur indirectly through co-regulators or mechanisms analogous to the effects of short-range repressors on nearby activators [64]. Importantly, other counter-repressors have been identified in *Drosophila melanogaster* development. Stat92E functions as a counter-repressor in the formation of the posterior spiracle, but its target repressor remains unknown [65]. Different counter repressors may exert their effects through different molecular mechanisms.

A long-term goal of studying gene regulation is predicting the output of a specific enhancer [10]. Context-dependent TF function [e.g. [66]] presents a challenge for meeting this goal. Here, we further elucidate counter-repression, one type of context dependence, and offer some strategies for uncovering it in regulatory DNA. First, single mutations of annotated activators or bifunctional TFs may be misleading. Analyzing the effects of single and double mutations together are necessary to classify activators, repressors, counter-repressors and the targets of counter-repression. For example, let’s say that you mutate binding sites for TF #1 and expression decreases. TF #1 may either be an activator or a counter-repressor. You now combine mutations in TF #1 with mutations in TF #2, and expression is restored. TF #2 may be a repressor or the target of counter-repression. Finally, you examine the effect of mutating TF #2 alone. If TF #2 is a repressor, expression will increase, but if TF #2 is the target of counter-repression, there will be no effect. All three experiments are necessary to correctly classify both TF #1 and TF #2. High-throughput mutational studies both in reporters [9] and *in vivo* [67] may be able to systematically gather this type of data on combinatorial effects.

### Conclusion

By combining targeted genetic perturbations with quantitative expression measurements, we uncovered counter-repression of Hb as a key feature of *eve* stripe 2 regulation. These results emphasize the complexity of animal developmental enhancers, and argue that the current textbook model of *eve* stripe 2 regulation is incomplete. We suggest that counter-repression increases the flexibility in the regulatory logic of *eve* stripe 2, which may impact its plasticity over evolutionary time.

## Materials and methods

### Binding site predictions and construct design

We used PATSER [68] to predict binding sites in *eve3+7* and *eve2+7* for blastoderm TFs. We used position weight matrices (PWMs) derived from bacterial 1-hybrid experiments for the following factors: Bicoid, Caudal, Dichaete, Stat92E, Hunchback, Krüppel, and Nubbin [69]. We used other published PWMs for Giant, Knirps, and Tailless [70]. Finally, we used a Zelda PWM from a personal communication with Christine Rushlow. Count matrices were converted into frequency matrices for use in PATSER using a pseudocount of 1. In designing binding site mutations, we predicted the effects on all predicted sites above a p-value cutoff of 0.003. In an effort to minimize impacts on other binding sites, we chose to leave some low affinity sites unmutated in both *eve3+7* and *eve2+7*. Annotated sequences of wild-type and mutated versions of enhancers can be found in S1 Appendix (*eve3+7* and *eve2+7* enhancers) and S2 Appendix (*eve* stripe 2 enhancers). We have posted csv files containing binding site predictions for all enhancers on Figshare, along with the frequency matrices we used for designing mutations (https://figshare.com/articles/Quantitative_Data_and_Analyses_for_Vincent_et_al_2018/6233600).

### Fly work

All reporter constructs were cloned into the NotI/BglII insertion site in the pBΦY reporter plasmid [51] and integrated into the attP2 landing site using injection services provided by BestGene Inc [71]. Successful transformants were homozygosed using the mini-white marker. We generated *sna*::*hb* embryos as described previously [24] by crossing virgin females containing a male germline-specific β2-tubulin–FLP transgene with males containing two copies of the *sna*::*hb* transgene flanked by FRT sites, both generous gifts from Steve Small. F1 males were then crossed to virgin females homozygous for the enhancer reporter construct of interest. We generated *cad* RNAi embryos as described previously [50] by crossing virgin females containing a *mat-tub-GAL4* driver with males containing short hairpin RNAs for *cad* driven by an upstream activating sequence (Bloomington Stock #34702). F1 virgin females were then crossed to males containing *eve2+7* reporter constructs to generate *cad* RNAi embryos.

### *In situ* hybridization and data analysis

Embryos were collected, fixed in heptane and paraformaldehyde, stained using quantitative *in situ* hybridization, staged into 6 timepoints using membrane invagination as a morphological marker, imaged using 2-photon confocal microscopy and processed to extract cellular resolution expression measurements as previously described [42,72]. For each embryo, we measure *fushi-tarazu* mRNA using an anti-digoxigenin horseradish peroxidase (HRP) antibody (Roche) and a coumarin-tyramide color reaction (PerkinElmer), as well as *lacZ* mRNA using an anti-2,4-dinitrophenyl HRP antibody (PerkinElmer) and a Cy3-tyramide color reaction (PerkinElmer). The *sna*::*hb* crossing scheme generates wild-type and mutant embryos in approximately equal proportions; we identified embryos misexpressing *hb* using the *ftz* expression pattern. For individual genotypes, we use a previously described computational pipeline to average measurements from multiple embryos together to generate gene expression atlases [43], which we display as 2-dimensional projections in this study. For atlas building, we used the coarse registration method for all genotypes because we lacked a *ftz* expression template for *sna*::*hb* embryos. To quantify the effect of ventral Hb in individual embryos, we normalized cellular expression measurements by the maximum value and subtracted the peak stripe expression value along the lateral side from the peak value along the ventral side. For experiments involving a *hkb* co-stain, we stained embryos in the same batch, discarded outliers, and ensured that *hkb* levels and *lacZ* levels were significantly correlated as described previously [44,52]. Means and standard errors for our quantitative measurements can be found in S1 and S2 Tables. We have deposited the processed expression data for all embryos, which we call pointclouds, along with the MATLAB code used to generate all data figures in this study on Figshare (see link above).

### Binding site enrichment analysis

Binding site enrichment was calculated as described previously [52]. In summary, we used PWMs from FlyFactorSurvey [61] to predict binding sites in orthologous *eve* enhancers, and compared that result with a null expectation for an enhancer of identical size that we generated by predicting sites in accessible areas of the genome during state 5 of embryogenesis [73]. This method depends on the PATSER score threshold used to identifying functional binding sites, which is difficult to define rigorously. We therefore calculated enrichment scores at all integer values for this threshold between 1 and the maximum score for the PWM; this method amounts to a sensitivity analysis for the PATSER score threshold. We have deposited our binding site predictions for all orthologous *eve* enhancers in csv format along with the MATLAB code used to calculate enrichment scores on Figshare (see link above).

## Supporting information

S1 Fig Quantitative effects of *eve3+7* mutations designed by Struffi et al. (A) Predicted Hb binding sites (blue) in *eve3+7* and *eve3+7mutHb* are plotted as in Fig 2. *eve3+7mutHb* sequence was taken from [26]. (B) 2D projections of atlas data for reporter constructs expressed in WT or *sna*::*hb* embryos. Data is taken from timepoint 4 (25–50% membrane invagination). Low-level anterior and posterior expression is due to an unused *hkb* co-stain. (C) Lateral line traces from individual wild-type embryos containing *eve3+7* reporter constructs (WT: grey, n = 26; m*utHb*: blue, n = 11). Each trace is normalized to its maximum value. Embryos are from all six timepoints in stage 5. (D) Differences in the maximum values of ventral and lateral line traces are plotted for individual wild-type and *sna*::*hb* embryos containing *eve3+7mutHb* in all 6 timepoints in stage 5. wt: n = 11; *sna*::*hb*: n = 12. Differences between wild-type and *sna*::*hb* embryos were not significant (Mann-Whitney U test, p-value > 0.1 for both stripes).
(TIF)

S2 Fig Line traces for individual embryos containing *eve2+7* reporter constructs. Wild-type: grey, n = 47; *mutHb*: cerulean, n = 42; *mutCad*: salmon, n = 35; *mutCadmutHb*: mauve, n = 32. Each trace is normalized to its maximum value. Embryos are from all six timepoints in stage 5. Expression anterior to 90% and posterior to 5% AP length is not shown and may be partially due to an unused *huckebein* co-stain in some embryos.
(TIF)

S3 Fig Hunchback mutations have different effects depending on enhancer context. (A) Predicted binding sites for Hb (blue) and other *eve2+7* regulators (grey) in different *eve* stripe 2 enhancer constructs (46,47). (B) Peak stripe 2 expression levels for individual embryos from timepoints 2–4 (4–50% membrane invagination) were measured using a *hkb* co-stain method (44). Asterisks indicate p-values < 0.05 (Mann-Whitney U test). Note that because each experiment was performed in separate hybridizations, comparisons can only be made between wild-type and mutated versions of the same enhancer, not between different stripe 2 enhancers.
(TIF)

S4 Fig Cad mutations may have disrupted one or more Giant binding sites. Predicted binding for Cad (red) and Gt (lilac) are shown in *eve2+7* and *eve2+7mutCad*. Many predicted Giant binding sites are near predicted Cad sites. One Cad binding site mutation in *eve2+7mutCad* (grey box) disrupts a predicted Gt binding site that also overlaps an annotated Gt binding site [47].
(TIF)

S5 Fig *eve* expression is not perturbed in *cad* RNAi embryos. 2D projections of *eve* expression data from representative wild-type (left) and *cadRNAi* embryos. Both embryos were from timepoint 3 (15% membrane invagination).
(TIF)

S6 Fig Caudal directly activates *eve3+7*. (A) Predicted Cad binding sites in *eve3+7* and *eve3+7mutCad*. Sites were predicted and displayed as described in previous figures. (B) Lateral line traces from individual wild-type embryos containing reporter constructs for *eve3+7* (grey, n = 11) and *eve3+7mutCad* (red, n = 7). Traces were normalized using a co-stain method [44]; embryos are from timepoints 3 and 4 (9–50% membrane invagination). (C) Individual stripe peaks were found by taking local maxima from line traces in B. Asterisks indicate significant differences in stripe level (p-values < 0.001, Mann-Whitney U test).
(TIF)

S7 Fig Sensitivity analyses for Caudal and Hunchback enrichment correlations. (A) Spearman correlation values (rho) of Cad and Hb binding site enrichment scores are plotted as a function of binding site threshold for *eve2* and *eve3+7* orthologs. Binding site threshold refers to the minimum PATSER score for a predicted site to be counted in the analysis. Higher PATSER scores are assumed to reflect higher affinity sites. (B) Hb and Cad enrichment values are plotted for individual *eve2* and *eve3+7* orthologs at a binding site threshold of 7.2 –the threshold that maximizes rho in *eve3+7* orthologs.
(TIF)

S8 Fig Sensitivity analyses for Bicoid and Hunchback enrichment correlations. Spearman correlation values (rho) of Bcd and Hb binding site enrichment scores are plotted as a function of binding site threshold for *eve2* (left) and *eve3+7* (right) orthologs. Enrichment scores for Bcd and Hb sites are not significantly correlated at any binding site threshold in either *eve2* or *eve3+7* orthologs.
(TIF)

S1 Appendix *eve3+7* and *eve2+7* enhancer sequences. Here, we list the sequences for the *eve3+7* and *eve2+7* reporter constructs used in the study. Predicted Hb binding sites are indicated in blue, and predicted Cad binding sites are indicated in red. We have indicated any base pairs that are shared by both a Hb binding site and a Cad binding site in purple. As detailed in the Materials and Methods, we did not mutate all predicted sites for each factor because we tried to minimize the impact on previously annotated or predicted sites for other *eve* regulators. PATSER scores for all predicted sites in each enhancer can be found on Figshare (see link above).
(DOCX)

S2 Appendix *eve* stripe 2 enhancer sequences. Here, we have listed the sequences for *eve* stripe 2 reporter constructs included in our study. Previously identified Hb binding sites are indicated in blue [46].
(DOCX)

S1 Table Means and standard errors of ventral and lateral peak expression comparisons for *eve* reporter constructs. We quantify the effect of ventral Hb by comparing the difference in ventral and lateral peak expression levels between wild-type and *sna*::*hb* embryos (see Fig 2 and Materials and Methods).
(XLSX)

S2 Table Means and standard errors of relative stripe levels driven by *eve* reporter constructs. We compare relative stripe levels between embryos expressing different reporter constructs by normalizing *lacZ* expression using a *huckebein* co-stain [44,52]. Using this method, only comparisons between embryos stained in the same hybridization batch are meaningful. Samples contained in the same batch are grouped together in the table.
(XLSX)

S3 Table Krüppel enrichment scores in orthologous *eve* enhancers. We calculated Kr enrichment scores for orthologs using two different PATSER score thresholds. Positive values indicate an overrepresentation in Kr binding sites compared to a null model, while negative values indicate underrepresentation. In general, Kr binding sites are enriched in *eve2* orthologs and poorly enriched or depleted in *eve3+7* and *eve4+6* orthologs. *Kr* is a repressor that is thought to define the posterior border of stripe 2 [14,47], but overlaps the expression patterns driven by *eve3+7* and *eve4+6* [15,16].
(XLSX)
