# Supplementary figures and images for: Hunchback is counter-repressed to regulate even-skipped stripe 2 expression in Drosophila embryos
Source: PLoS Genet. 2018 Sep 7;14(9):e1007644. doi: 10.1371/journal.pgen.1007644 (PMC6145585; doi:10.1371/journal.pgen.1007644)

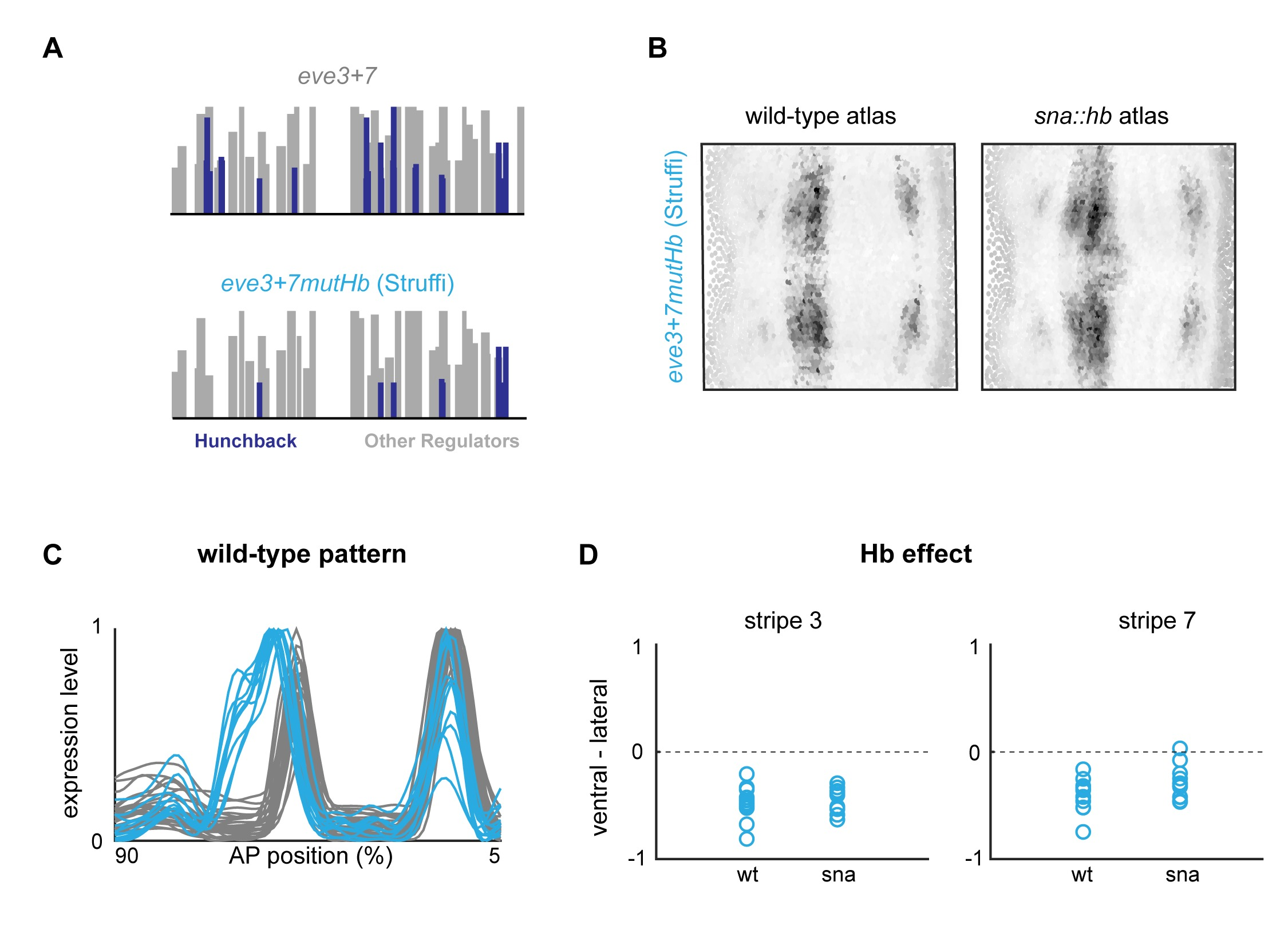

Supplement: S1 Fig — (A) Predicted Hb binding sites (blue) in eve3+7 and eve3+7mutHb are plotted as in Fig 2. eve3+7mutHb sequence was taken from [26]. (B) 2D projections of atlas data for reporter constructs expressed in WT or sna::hb embryos. Data is taken from timepoint 4 (25–50% membrane invagination). Low-level anterior and posterior expression is due to an unused hkb co-stain. (C) Lateral line traces from individual wild-type embryos containing eve3+7 reporter constructs (WT: grey, n = 26; mutHb: blue, n = 11). Each trace is normalized to its maximum value. Embryos are from all six timepoints in stage 5. (D) Differences in the maximum values of ventral and lateral line traces are plotted for individual wild-type and sna::hb embryos containing eve3+7mutHb in all 6 timepoints in stage 5. wt: n = 11; sna::hb: n = 12. Differences between wild-type and sna::hb embryos were not significant (Mann-Whitney U test, p-value > 0.1 for both stripes). (TIF) [file pgen.1007644.s001.tif]

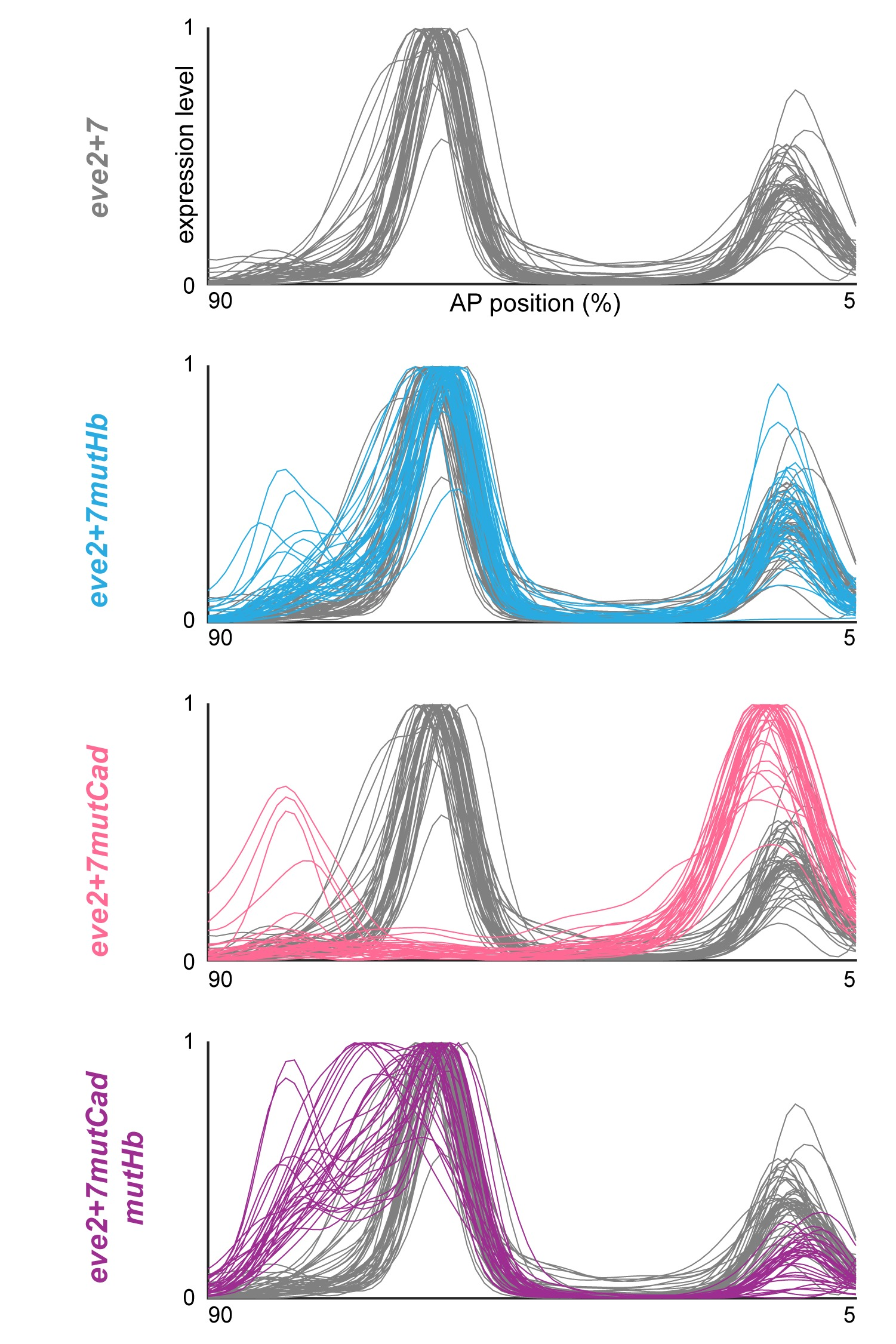

Supplement: S2 Fig — Wild-type: grey, n = 47; mutHb: cerulean, n = 42; mutCad: salmon, n = 35; mutCadmutHb: mauve, n = 32. Each trace is normalized to its maximum value. Embryos are from all six timepoints in stage 5. Expression anterior to 90% and posterior to 5% AP length is not shown and may be partially due to an unused huckebein co-stain in some embryos. (TIF) [file pgen.1007644.s002.tif]

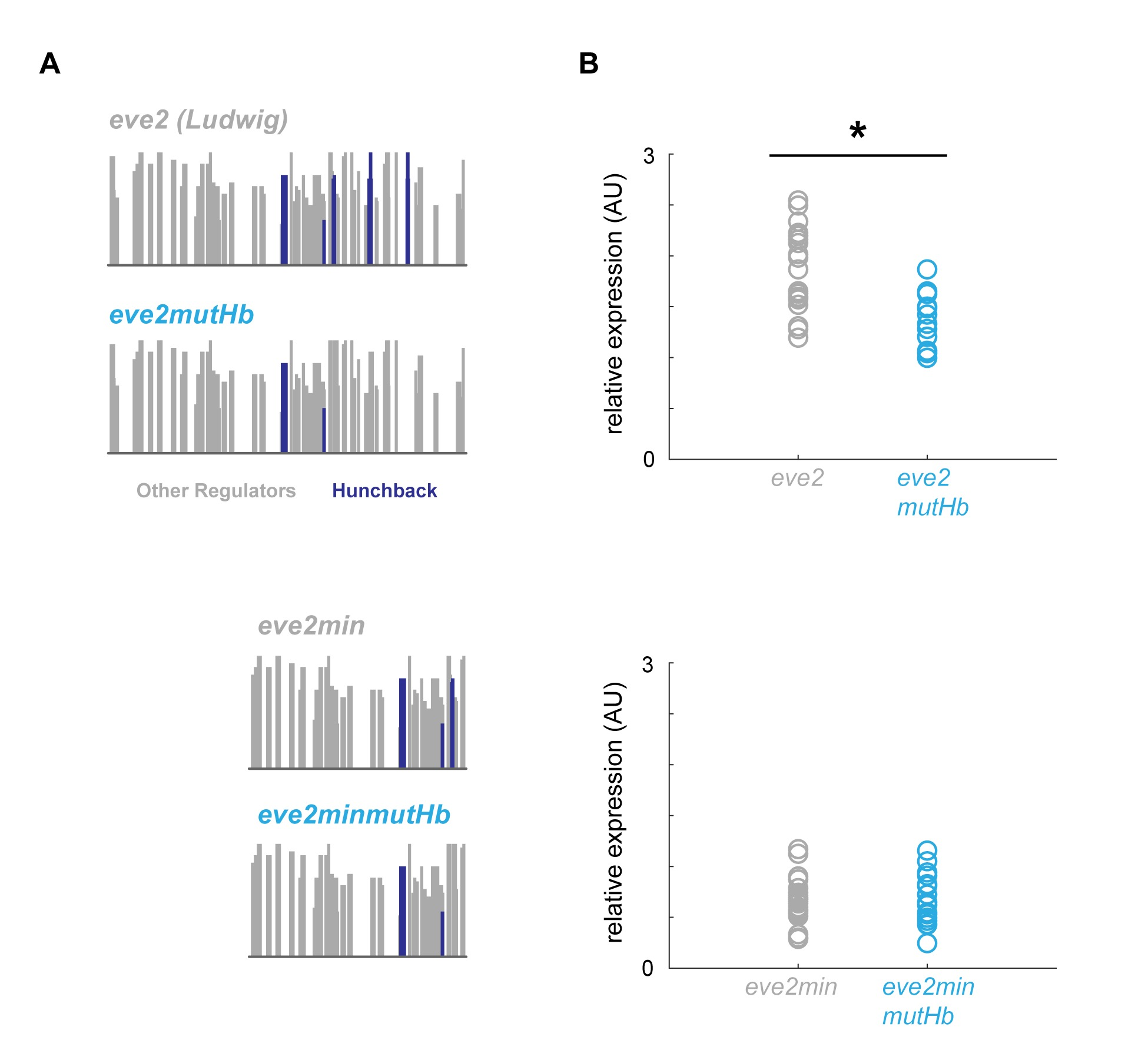

Supplement: S3 Fig — (A) Predicted binding sites for Hb (blue) and other eve2+7 regulators (grey) in different eve stripe 2 enhancer constructs (46,47). (B) Peak stripe 2 expression levels for individual embryos from timepoints 2–4 (4–50% membrane invagination) were measured using a hkb co-stain method (44). Asterisks indicate p-values < 0.05 (Mann-Whitney U test). Note that because each experiment was performed in separate hybridizations, comparisons can only be made between wild-type and mutated versions of the same enhancer, not between different stripe 2 enhancers. (TIF) [file pgen.1007644.s003.tif]

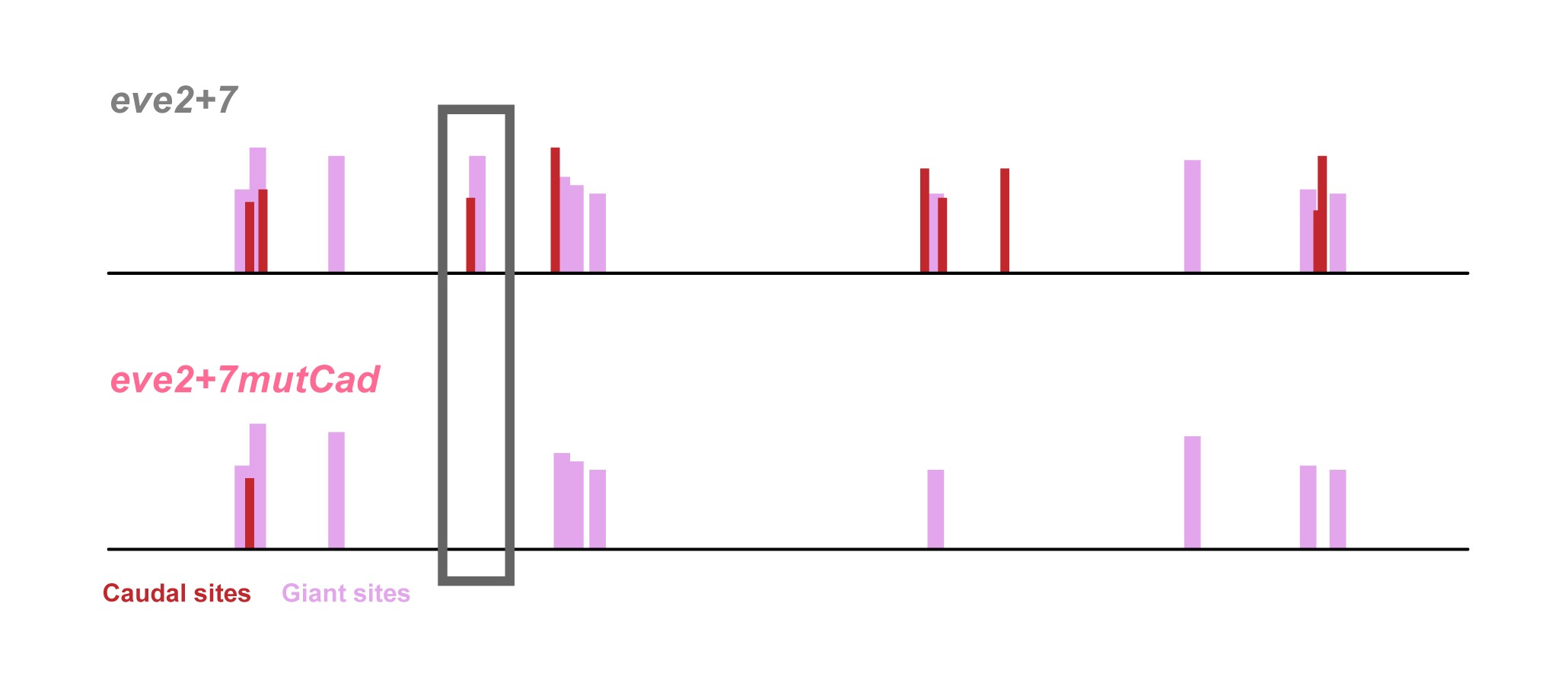

Supplement: S4 Fig — Predicted binding for Cad (red) and Gt (lilac) are shown in eve2+7 and eve2+7mutCad. Many predicted Giant binding sites are near predicted Cad sites. One Cad binding site mutation in eve2+7mutCad (grey box) disrupts a predicted Gt binding site that also overlaps an annotated Gt binding site [47]. (TIF) [file pgen.1007644.s004.tif]

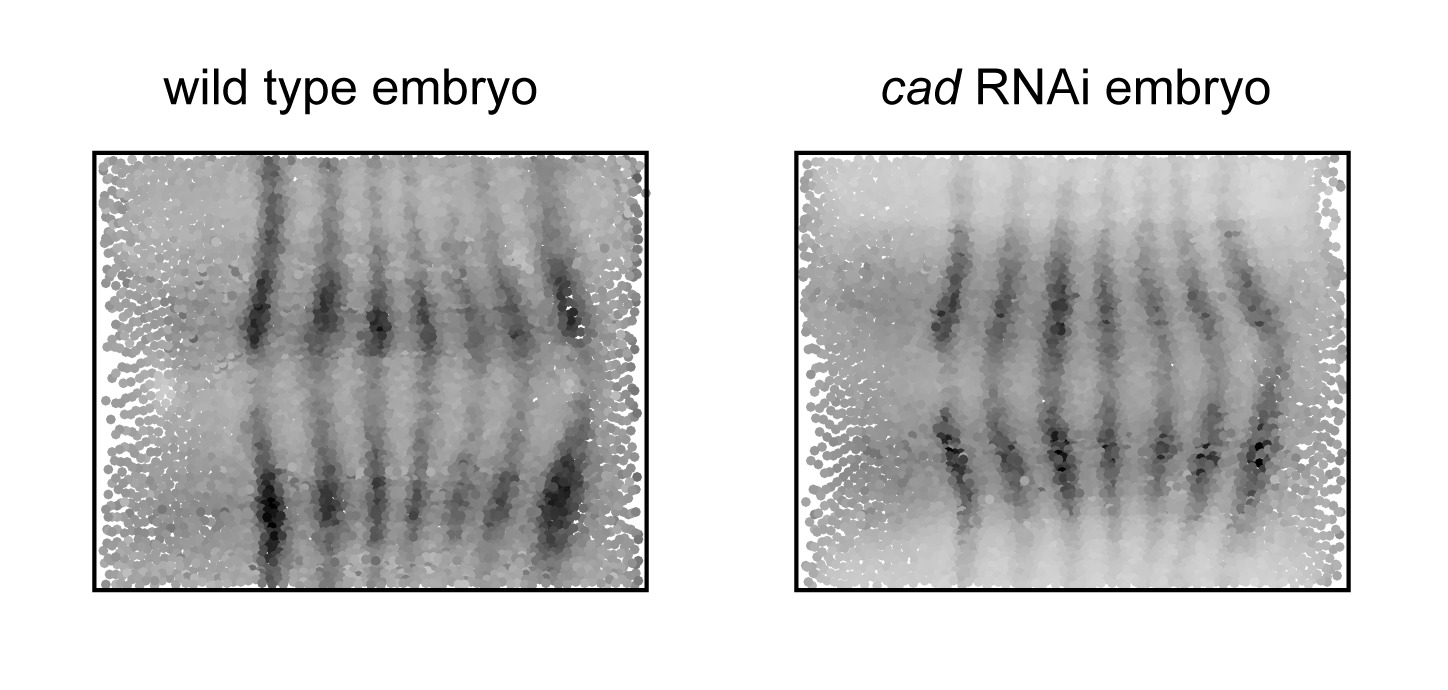

Supplement: S5 Fig — 2D projections of eve expression data from representative wild-type (left) and cadRNAi embryos. Both embryos were from timepoint 3 (15% membrane invagination). (TIF) [file pgen.1007644.s005.tif]

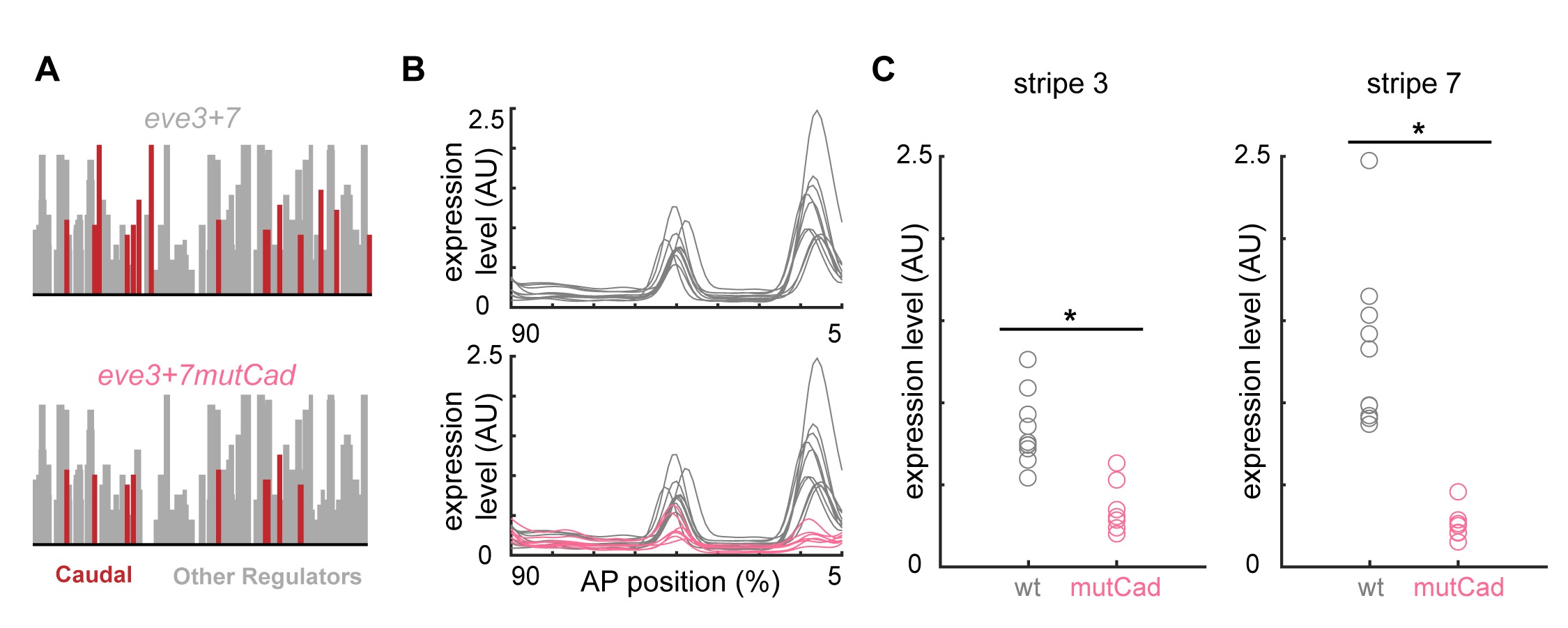

Supplement: S6 Fig — (A) Predicted Cad binding sites in eve3+7 and eve3+7mutCad. Sites were predicted and displayed as described in previous figures. (B) Lateral line traces from individual wild-type embryos containing reporter constructs for eve3+7 (grey, n = 11) and eve3+7mutCad (red, n = 7). Traces were normalized using a co-stain method [44]; embryos are from timepoints 3 and 4 (9–50% membrane invagination). (C) Individual stripe peaks were found by taking local maxima from line traces in B. Asterisks indicate significant differences in stripe level (p-values < 0.001, Mann-Whitney U test). (TIF) [file pgen.1007644.s006.tif]

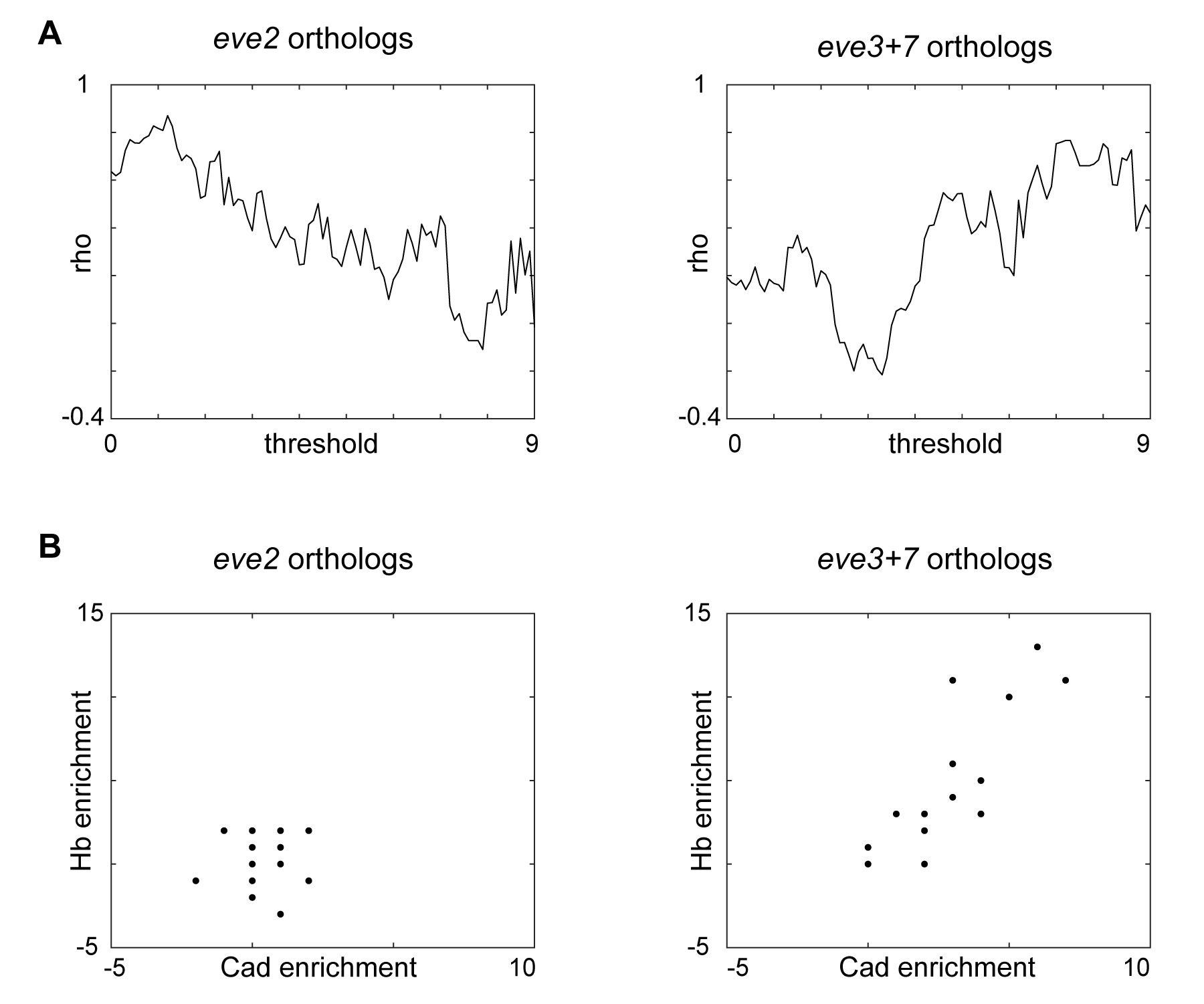

Supplement: S7 Fig — (A) Spearman correlation values (rho) of Cad and Hb binding site enrichment scores are plotted as a function of binding site threshold for eve2 and eve3+7 orthologs. Binding site threshold refers to the minimum PATSER score for a predicted site to be counted in the analysis. Higher PATSER scores are assumed to reflect higher affinity sites. (B) Hb and Cad enrichment values are plotted for individual eve2 and eve3+7 orthologs at a binding site threshold of 7.2 –the threshold that maximizes rho in eve3+7 orthologs. (TIF) [file pgen.1007644.s007.tif]

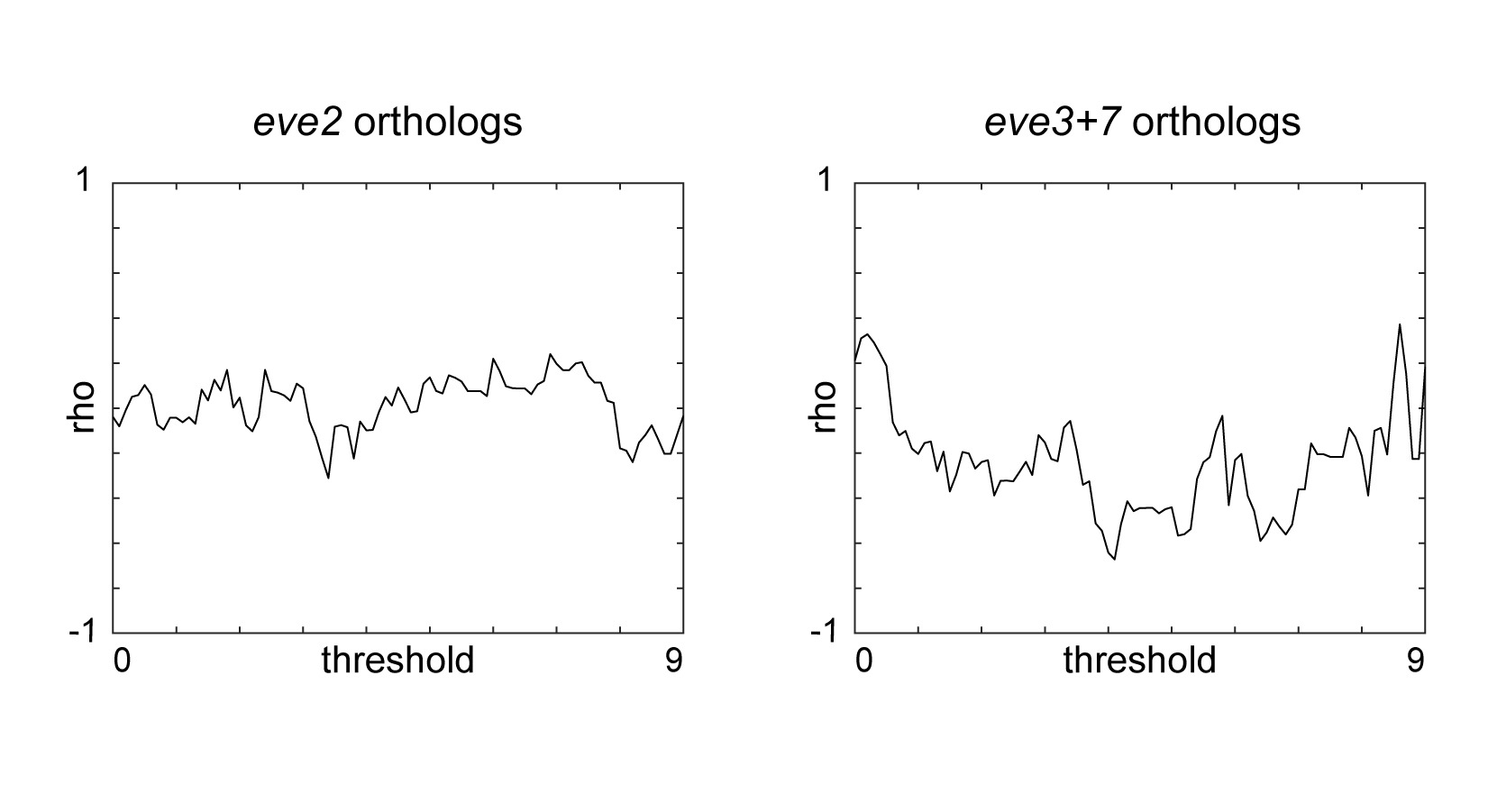

Supplement: S8 Fig — Spearman correlation values (rho) of Bcd and Hb binding site enrichment scores are plotted as a function of binding site threshold for eve2 (left) and eve3+7 (right) orthologs. Enrichment scores for Bcd and Hb sites are not significantly correlated at any binding site threshold in either eve2 or eve3+7 orthologs. (TIF) [file pgen.1007644.s008.tif]
