# Supplementary material for: Hunchback is counter-repressed to regulate even-skipped stripe 2 expression in Drosophila embryos
Source: PLoS Genet. 2018 Sep 7;14(9):e1007644. doi: 10.1371/journal.pgen.1007644 (PMC6145585; doi:10.1371/journal.pgen.1007644)
Supplement: S1 Appendix — Here, we list the sequences for the eve3+7 and eve2+7 reporter constructs used in the study. Predicted Hb binding sites are indicated in blue, and predicted Cad binding sites are indicated in red. We have indicated any base pairs that are shared by both a Hb binding site and a Cad binding site in purple. As detailed in the Materials and Methods, we did not mutate all predicted sites for each factor because we tried to minimize the impact on previously annotated or predicted sites for other eve regulators. PATSER scores for all predicted sites in each enhancer can be found on Figshare (see link above). (DOCX) [file pgen.1007644.s009.docx]

>*eve3+7*, DePace stock #0204

GGATCCTCGAAATCGAGAGCGACCTCGCTGCATTAGAAAACTAGATCAGTTTTTTGTTTTGGCCGACCGATTTTTGTGCCCGGTGCTCTCTTTACGGTTTATGGCCGCGTTCCCATTTCCCAGCTTCTTTGTTCCGGGCTCAGAAATCTGTATGGAATTATGGTATATGCAGATTTTTATGGGTCCCGGCGATCCGGTTCGCGGAACGGGAGTGTCCTGCCGCGAGAGGTCCTCGCCGGCGATCCTTGTCGCCCGTATTAGGAAAGTAGATCACGTTTTTTGTTCCCATTGTGCGCTTTTTTCGCTGCGCTAGTTTTTTTCCCCGAACCCAGCGAACTGCTCTAATTTTTTAATTCTTCACGGCTTTTCATTGGGCTCCTGGAAAAACGCGGACAAGGTTATAACGCTCTACTTACCTGCAATTGTGGCCATAACTCGCACTGCTCTCGTTTTTAAGATCCGTTTGTTTGTGTTTGTTTGTCCGCGATGGCATTCACGTTTTTACGAGCTC

>*eve3+7mutHb*, DePace stock #0506

GGATCCTCGAAATCGAGAGCGACCTCGCTGCATTAGAAAACTAGATCAGTTccTTGTTTTGGCCGACCGATccTTGTGCCCGGTGCTCTCTTTACGGTTTATGGCCGCGTTCCCATTTCCCAGCTTCTTTGTTCCGGGCTCAGAAATCTGTATGGAATTATGGTATATGCAGAccTTTATGGGTCCCGGCGATCCGGTTCGCGGAACGGGAGTGTCCTGCCGCGAGAGGTCCTCGCCGGCGATCCTTGTCGCCCGTATTAGGAAAGTAGATCACGTTccTTGTTCCCATTGTGCGCTTccTTCGCTGCGCTAGTTccTTTCCCCGAACCCAGCGAACTGCTCTAATcggTcAATTCTTCACGGCTTTTCATTGGGCTCCTGGAAAAACGCGGACAAGGTTATAACGCTCTACTTACCTGCAATTGTGGCCATAACTCGCACTGCTCTCGTTTTTAAGATCCGTTTGTTTGTGTTTGTTTGTCCGCGATGGCATTCACGTTTTTACGAGCTC

>*eve3+7mutHb* (Struffi construct), DePace stock #0507

GGATCCTCGAAATCGAGAGCGACCTCGCTGCATTAGAAAACTAGATCAGTcTTcTaTTTTGGCCGACCGATTcTTaTaCCCGGTGCTCTCTTTACGGTTTATGGCCGCGTTCCCATTTCCCAGCTTCTTTGTTCCGGGCTCAGAAATCTGTATGGAATTATGGTATATGCAGATTcTTATaGGTCCCGGCGATCCGGTTCGCGGAACGGGAGTGTCCTGCCGCGAGAGGTCCTCGCCGGCGATCCTTGTCGCCCGTATTAGGAAAGTAGATCACGTTTTccGTTCCCATTGTGCGCTTcTTTCaCTGCGCTAGTTccTTTCCCCGAACCCAGCGAACTGCTCTAATTTccTAATTCTTCACGGCTTTTCATTGGGCTCCTGGAAAAACGCGGACAAGGTTATAACGCTCTACTTACCTGCAATTGTGGCCATAACTCGCACTGCTCTCGTTcTcAAGATCCGTTTGTTTGTGTTTGTTTGTCCGCGATGGCATTCACGTTcTTACaAGCTC

>*eve3+7mutCad*, DePace stock #0472

GGATCCTCGAAATCGAGAGCGACCTCGCTGCATTAGAAAACTAGATCAGTTTTTTGTTTTGGCCGACCGATTTTTGTGCCCGGTGCTCTCTTTACGGTgTcgGGCCGCGTTCCCATTTCCCAGCTTCTTTGTTCCGGGCTCAGAAATCTGTATGGAAgTcgGGTATATGCAGATTTgTcgGGGTCCCGGCGATCCGGTTCGCGGAACGGGAGTGTCCTGCCGCGAGAGGTCCTCGCCGGCGATCCTTGTCGCCCGTATTAGGAAAGTAGATCACGTTTTTTGTTCCCATTGTGCGCTTTTTTCGCTGCGCTAGTTTTTTTCCCCGAACCCAGCGAACTGCTCTAATTTTTTAATTCTTCACGGCTTTTCATTGGGCTCCTGGAAAAACGCGGACAAGGTTATAACGCTCTACTTACCTGCAATTGTGGCCcgAcCTCGCACTGCTCTCGTTTgTcgGATCCGTTTGTTTGTGTTTGTTTGTCCGCGATGGCATTCACGTTTcTgCGAGCTC

>*eve2+7*, DePace stock #0547

agaaggcttgcatgtgggccttttccaggtcggccagtaggtagagttgttgcgatgcggctatgccgggcgagttaatgccaatgcaaattgcgggcgcaatataacccaataatttgaagtaactggcaggagcgaggtatccttcctggttacccggtactgcataacaatggaacccgaaccgtaactgggacagatcgaaaagctggcctggtttctcgctgtgtgtgccgtgttaatccgtttgccatcagcgagattattagtcaattgcagttgcagcgtttcgctttcgtcctcgtttcactttcgagttagactttattgcagcatcttgaacaatcgtcgcagtttggtaacacgctgtgccatactttcatttagacggaatcgagggaccctggactataatcgcacaacgagaccgggttgcgaagtcagggcattccgccgatctagccatcgccatcttctgcgggcgtttgtttgtttgtttgctgggattagccaagggcttgacttggaatccaatcccgatccctagcccgatcccaatcccaatcccaatcccttgtccttttcattagaaagtcataaaaacacataataatgatgtcgaagggattaggggcgcgcaggtccaggcaacgcaattaacggactagcgaactgggttatttttttgcgccgacttagccctgatccgcgagcttaacccgttttgagccgggcagcaggtagttgtgggtggaccccacgatttttttggccaaacctccaagctaacttgcgcaagtggcaagtggccggtttgctggcccaaaagaggaggcactatcccggtcctggtacagttggtacgctgggaatgattatatcatcataataaatgttttgcccaacgaaaccgaaaacttttcaaattaagtcccggcaactgggttcccattttccattttccatgttctgcgggcaggggcggccattatctcgct

>*eve2+7 mut Hb*, DePace stock #0535

agaaggcttgcatgtgggccttttccaggtcggccagtaggtagagttgttgcgatgcggctatgccgggcgagttaatgccaatgcaaattgcgggcgcaatataacccaataatttgaagtaactggcaggagcgaggtatccttcctggttacccggtactgcataacaatggaacccgaaccgtaactgggacagatcgaaaagctggcctggtttctcgctgtgtgtgccgtgttaatccgtttgccatcagcgagattattagtcaattgcagttgcagcgtttcgctttcgtcctcgtttcactttcgagttagactttattgcagcatcttgaacaatcgtcgcagtttggtaacacgctgtgccatactttcatttagacggaatcgagggaccctggactataatcgcacaacgagaccgggttgcgaagtcagggcattccgccgatctagccatcgccatcttctgcgggcgtttgtttgtttgtttgctgggattagccaagggcttgacttggaatccaatcccgatccctagcccgatcccaatcccaatcccaatcccttgtccttttcattagaaagtcataaaGGcacataataatgatgtcgaagggattaggggcgcgcaggtccaggcaacgcaattaacggactagcgaactgggttattCCtttgcgccgacttagccctgatccgcgagcttaacccgttttgagccgggcagcaggtagttgtgggtggaccccacgattCCtttggccaaacctccaagctaacttgcgcaagtggcaagtggccggtttgctggcccaaaagaggaggcactatcccggtcctggtacagttggtacgctgggaatgattatatcatcataataaatgttttgcccaacgaaaccgaaaacttttcaaattaagtcccggcaactgggttcccattttccattttccatgttctgcgggcaggggcggccattatctcgct

>*eve2+7 mut Cad*, DePace stock #0549

agaaggcttgcatgtgggccttttccaggtcggccagtaggtagagttgttgcgatgcggctatgccgggcgagttaatgccaatgcaaattgcgggcgcaatataacccaataGGttgaagtaactggcaggagcgaggtatccttcctggttacccggtactgcataacaatggaacccgaaccgtaactgggacagatcgaaaagctggcctggtttctcgctgtgtgtgccgtgttaatccgtttgccatcagcgagattatATgtcaattgcagttgcagcgtttcgctttcgtcctcgtttcactttcgagttagacGCtattgcagcatcttgaacaatcgtcgcagtttggtaacacgctgtgccatactttcatttagacggaatcgagggaccctggactataatcgcacaacgagaccgggttgcgaagtcagggcattccgccgatctagccatcgccatcttctgcgggcgtttgtttgtttgtttgctgggattagccaagggcttgacttggaatccaatcccgatccctagcccgatcccaatcccaatcccaatcccttgtccttttcattagaaagtcGCaaaaacacataataGGgatgtcgaagggattaggggcgcgcaggtccaggcaacgcaGCtaacggactagcgaactgggttatttttttgcgccgacttagccctgatccgcgagcttaacccgttttgagccgggcagcaggtagttgtgggtggaccccacgatttttttggccaaacctccaagctaacttgcgcaagtggcaagtggccggtttgctggcccaaaagaggaggcactatcccggtcctggtacagttggtacgctgggaatgattatatcatcataGCaaatgttttgcccaacgaaaccgaaaacttttcaaattaagtcccggcaactgggttcccattttccattttccatgttctgcgggcaggggcggccattatctcgct

>*eve2+7 mut Cad and Hb*, DePace stock #0634

agaaggcttgcatgtgggccttttccaggtcggccagtaggtagagttgttgcgatgcggctatgccgggcgagttaatgccaatgcaaattgcgggcgcaatataacccaataGGttgaagtaactggcaggagcgaggtatccttcctggttacccggtactgcataacaatggaacccgaaccgtaactgggacagatcgaaaagctggcctggtttctcgctgtgtgtgccgtgttaatccgtttgccatcagcgagattatATgtcaattgcagttgcagcgtttcgctttcgtcctcgtttcactttcgagttagacGCtattgcagcatcttgaacaatcgtcgcagtttggtaacacgctgtgccatactttcatttagacggaatcgagggaccctggactataatcgcacaacgagaccgggttgcgaagtcagggcattccgccgatctagccatcgccatcttctgcgggcgtttgtttgtttgtttgctgggattagccaagggcttgacttggaatccaatcccgatccctagcccgatcccaatcccaatcccaatcccttgtccttttcattagaaagtcGCaaaGGcacataataGGgatgtcgaagggattaggggcgcgcaggtccaggcaacgcaGCtaacggactagcgaactgggttattCCtttgcgccgacttagccctgatccgcgagcttaacccgttttgagccgggcagcaggtagttgtgggtggaccccacgattCCtttggccaaacctccaagctaacttgcgcaagtggcaagtggccggtttgctggcccaaaagaggaggcactatcccggtcctggtacagttggtacgctgggaatgattatatcatcataGCaaatgttttgcccaacgaaaccgaaaacttttcaaattaagtcccggcaactgggttcccattttccattttccatgttctgcgggcaggggcggccattatctcgct
