# Supplementary material for: Hunchback is counter-repressed to regulate even-skipped stripe 2 expression in Drosophila embryos
Source: PLoS Genet. 2018 Sep 7;14(9):e1007644. doi: 10.1371/journal.pgen.1007644 (PMC6145585; doi:10.1371/journal.pgen.1007644)
Supplement: S2 Appendix — Here, we have listed the sequences for eve stripe 2 reporter constructs included in our study. Previously identified Hb binding sites are indicated in blue [46]. (DOCX) [file pgen.1007644.s010.docx]

>*eve2* (Ludwig), DePace stock #0464

aatataacccaataatttgaagtaactggcaggagcgaggtatccttcctggttacccggtactgcataacaatggaacccgaaccgtaactgggacagatcgaaaagctggcctggtttctcgctgtgtgtgccgtgttaatccgtttgccatcagcgagattattagtcaattgcagttgcagcgtttcgctttcgtcctcgtttcactttcgagttagactttattgcagcatcttgaacaatcgtcgcagtttggtaacacgctgtgccatactttcatttagacggaatcgagggaccctggactataatcgcacaacgagaccgggttgcgaagtcagggcattccgccgatctagccatcgccatcttctgcgggcgtttgtttgtttgtttgctgggattagccaagggcttgacttggaatccaatcccgatccctagcccgatcccaatcccaatcccaatcccttgtccttttcattagaaagtcataaaaacacataataatgatgtcgaagggattaggggcgcgcaggtccaggcaacgcaattaacggactagcgaactgggttatttttttgcgccgacttagccctgatccgcgagcttaacccgttttgagccgggcagcaggtagttgtgggtggaccccacgatttttttggccaaacctccaagctaacttgcgcaagtggcaagtggccggtttgctggcccaaaagaggaggcactatcccggtcctggtacagttggtacgctgggaatgattatatcatcataataaatgttt

>*eve2* (Ludwig) *mut Hb*, DePace stock #0496

aatataacccaataatttgaagtaactggcaggagcgaggtatccttcctggttacccggtactgcataacaatggaacccgaaccgtaactgggacagatcgaaaagctggcctggtttctcgctgtgtgtgccgtgttaatccgtttgccatcagcgagattattagtcaattgcagttgcagcgtttcgctttcgtcctcgtttcactttcgagttagactttattgcagcatcttgaacaatcgtcgcagtttggtaacacgctgtgccatactttcatttagacggaatcgagggaccctggactataatcgcacaacgagaccgggttgcgaagtcagggcattccgccgatctagccatcgccatcttctgcgggcgtttgtttgtttgtttgctgggattagccaagggcttgacttggaatccaatcccgatccctagcccgatcccaatcccaatcccaatcccttgtccttttcattagaaagtcaGCaTCTcacataataatgatgtcgaagggattaggggcgcgcaggtccaggcaacgcaattaacggactagcgaactgggttatGCAtCtgcgccgacttagccctgatccgcgagcttaacccgttttgagccgggcagcaggtagttgtgggtggaccccacgatGCAtCtggccaaacctccaagctaacttgcgcaagtggcaagtggccggtttgctggcccaaaagaggaggcactatcccggtcctggtacagttggtacgctgggaatgattatatcatcataataaatgttt

>*eve2min*, DePace stock #0216

ggttacccggtactgcataacaatggaacccgaaccgtaactgggacagatcgaaaagctggcctggtttctcgctgtgtgtgccgtgttaatccgtttgccatcagcgagattattagtcaattgcagttgcagcgtttcgctttcgtcctcgtttcactttcgagttagactttattgcagcatcttgaacaatcgtcgcagtttggtaacacgctgtgccatactttcatttagacggaatcgagggaccctggactataatcgcacaacgagaccgggttgcgaagtcagggcattccgccgatctagccatcgccatcttctgcgggcgtttgtttgtttgtttgctgggattagccaagggcttgacttggaatccaatcccgatccctagcccgatcccaatcccaatcccaatcccttgtccttttcattagaaagtcataaaaacacataataatgatgtcgaagggattagggg

>*eve2min mut Hb*, DePace stock #0463

GgttacccggtactgcataacaatggaacccgaaccgtaactgggacagatcgaaaagctggcctggtttctcgctgtgtgtgccgtgttaatccgtttgccatcagcgagattattagtcaattgcagttgcagcgtttcgctttcgtcctcgtttcactttcgagttagactttattgcagcatcttgaacaatcgtcgcagtttggtaacacgctgtgccatactttcatttagacggaatcgagggaccctggactataatcgcacaacgagaccgggttgcgaagtcagggcattccgccgatctagccatcgccatcttctgcgggcgtttgtttgtttgtttgctgggattagccaagggcttgacttggaatccaatcccgatccctagcccgatcccaatcccaatcccaatcccttgtccttttcattagaaagtcaGCaTCTcacataataatgatgtcgaagggattagggg
